# Simple Equations Method (SEsM): An Effective Algorithm for Obtaining Exact Solutions of Nonlinear Differential Equations

**DOI:** 10.3390/e24111653

**Published:** 2022-11-14

**Authors:** Nikolay K. Vitanov

**Affiliations:** 1 Institute of Mechanics, Bulgarian Academy of Sciences, Acad. G. Bonchev Str., Bl. 4, 1113 Sofia, Bulgaria; vitanov@imbm.bas.bg; 2 Climate, Atmosphere and Water Research Institute, Bulgarian Academy of Sciences, Blvd. Tzarigradsko Chaussee 66, 1784 Sofia, Bulgaria

**Keywords:** nonlinear differential equations, solitons, exact solutions, simple equations method, modified method of the simplest equation, method of the inverse scattering transform, method of Hirota, Faa di Bruno formula for derivatives of a composite function, differential equation of Bernoulli, differential equation of Riccati, Korteweg-de Vries equation, nonlinear Schrödinger equation, Olver equation, SIR model of epidemic spreading, COVID-19, equation of fisher, equation of Burgers–Huxley, generalized equation of Camassa–Holm, generalized equation of Swift–Hohenberg, generalized Rayleigh equation, Jacobi elliptic function expansion method, f-expansion method, modified simple equation method, trial function method, general projective Riccati equations method, first integral method, homogeneous balance method, auxiliary equation method, G’/G method, exp-function method, tanh method

## Abstract

Exact solutions of nonlinear differential equations are of great importance to the theory and practice of complex systems. The main point of this review article is to discuss a specific methodology for obtaining such exact solutions. The methodology is called the SEsM, or the Simple Equations Method. The article begins with a short overview of the literature connected to the methodology for obtaining exact solutions of nonlinear differential equations. This overview includes research on nonlinear waves, research on the methodology of the Inverse Scattering Transform method, and the method of Hirota, as well as some of the nonlinear equations studied by these methods. The overview continues with articles devoted to the phenomena described by the exact solutions of the nonlinear differential equations and articles about mathematical results connected to the methodology for obtaining such exact solutions. Several articles devoted to the numerical study of nonlinear waves are mentioned. Then, the approach to the SEsM is described starting from the Hopf–Cole transformation, the research of Kudryashov on the Method of the Simplest Equation, the approach to the Modified Method of the Simplest Equation, and the development of this methodology towards the SEsM. The description of the algorithm of the SEsM begins with the transformations that convert the nonlinearity of the solved complicated equation into a treatable kind of nonlinearity. Next, we discuss the use of composite functions in the steps of the algorithms. Special attention is given to the role of the simple equation in the SEsM. The connection of the methodology with other methods for obtaining exact multisoliton solutions of nonlinear differential equations is discussed. These methods are the Inverse Scattering Transform method and the Hirota method. Numerous examples of the application of the SEsM for obtaining exact solutions of nonlinear differential equations are demonstrated. One of the examples is connected to the exact solution of an equation that occurs in the SIR model of epidemic spreading. The solution of this equation can be used for modeling epidemic waves, for example, COVID-19 epidemic waves. Other examples of the application of the SEsM methodology are connected to the use of the differential equation of Bernoulli and Riccati as simple equations for obtaining exact solutions of more complicated nonlinear differential equations. The SEsM leads to a definition of a specific special function through a simple equation containing polynomial nonlinearities. The special function contains specific cases of numerous well-known functions such as the trigonometric and hyperbolic functions and the elliptic functions of Jacobi, Weierstrass, etc. Among the examples are the solutions of the differential equations of Fisher, equation of Burgers–Huxley, generalized equation of Camassa–Holm, generalized equation of Swift–Hohenberg, generalized Rayleigh equation, etc. Finally, we discuss the connection between the SEsM and the other methods for obtaining exact solutions of nonintegrable nonlinear differential equations. We present a conjecture about the relationship of the SEsM with these methods.

## 1. Short Introduction and Overview of the Literature

Nature and human societies are full of complex systems. Atomic chains and lattices, biological systems, the dynamics of research groups, networks, social sciences, and economics are some of the many examples [1,2,3,4,5,6,7,8]. These complex systems require sophisticated research instruments and powerful computers for their study. The research on complex systems is complicated as many of these systems are nonlinear. Examples of this nonlinearity can be seen in fluid mechanics, solid-state physics, etc. [9,10,11,12,13]. Time-series analysis and models based on differential or difference equations are often used in the studies of nonlinear complex systems [14,15,16,17,18,19].

This review is focused on one of the aspects of the study of complex systems: the use of nonlinear differential equations for modeling such systems. The research on nonlinear differential equations has a long history [20,21,22,23,24,25,26]. In particular, nonlinear waves have been attracting the attention of researchers for many decades [27,28,29,30,31,32,33,34,35,36,37,38,39,40,41,42,43]. Several examples of the occurrence of such waves are:

- elastic media, specifically elastic crystals [44,45];
- fluids [46,47,48,49,50,51,52], especially the interaction of nonlinear waves and offshore structures [53], and plasmas [54];
- solids [55], for example, nonlinear acoustic waves in solids [56] or nonlinear spin waves in magnetic films [57];
- optics [58], especially nonlinear waves in waveguides [59];
- chemical systems [60].

Much attention has been devoted to the research of nonlinear waves in dispersive systems [61,62,63,64,65,66]. These waves often belong to a special class of nonlinear waves: the solitons [67,68,69,70,71,72,73,74,75,76,77,78,79,80,81,82,83,84,85,86,87,88,89,90,91,92,93,94,95,96]. Solitons are predicted and observed in many systems such as conducting polymers [97], plasmas [98,99], and helium films [100], and can be used, for example, in long-distance communications [101].

Below, we describe a methodology for obtaining exact solutions of nonlinear differential equations. The methodology is called the SEsM (Simple Equations Method). The methodology is a recent development in a long line of studies of methods for obtaining exact solutions of nonlinear differential equations. The classic approach is the Inverse Scattering Transform method. The basis for the Inverse Scattering Transform method was described in the paper by Gardner, Greene, Kruskal, and Miura [102]. An excellent introduction to the methodology is [103,104]. Additional results on the methodology can be found in [105]. Lax [106] proposed a general principle for associating the evolution of nonlinear equations with linear operators so that the eigenvalues of the linear operator are integrals of the nonlinear equation. We also note the contribution of Zakharov and Shabat [107,108] and the results of Fokas and Ablowitz [109,110]; Manakov [111,112]; Dod and Bullough [113]; Ablowitz, Kruskal, and Segur [114]; Kaup and Newell [115,116]; Beals [117]; Kaup [118,119]; and other authors [120,121]. This short list of contributors is in no way complete.

The Inverse Scattering Transform method stimulated much research on nonlinear differential equations. We have already mentioned above the nonlinear waves in dispersive systems and solitons. Additional results have been obtained on the integrability of dynamical systems [122,123] and the integrability of evolution equations [124,125,126,127,128,129,130,131], the construction of higher-dimensional integrable systems [132], the spectral transformations method for solving nonlinear evolution equations [133], etc. [134,135]. Other methods for obtaining exact solutions of nonlinear partial differential equations have also appeared. A prominent example of such a method is the method of Hirota [136,137,138,139,140,141].

Numerous equations have been studied using the above-mentioned methods. One of the most popular is the Korteweg-de Vries equation [142,143,144,145,146,147,148,149,150,151,152,153,154,155,156,157,158,159,160,161,162,163,164,165,166,167,168,169,170]. A short list of some other studied equations is as follows:

- sh-Gordon equation and Benney–Newell equation [171,172];
- sine-Gordon equation [173,174,175,176,177,178,179,180,181];
- nonlinear Schrödinger equation [182,183,184,185,186,187,188,189,190,191,192,193,194,195,196,197,198,199,200,201,202,203,204,205,206,207,208,209,210,211,212,213,214,215,216,217,218,219,220,221,222,223,224,225,226,227,228];
- Kuramoto–Sivashinsky equation [229] and Boussinesq equation [230,231,232,233];
- Pitaevskii–Gross equation [234] and Kadomtsev–Petviashvili equation [235,236];
- long-wave-short-wave resonance equations [237] and *N*-wave and Davey–Stewartson equations [238,239];
- Benjamin–Ono equation [240,241,242] and Camassa–Holm equation [243,244,245,246];
- long-wave equation [247,248] and Schamel equations [249];
- nonlinear Klein–Gordon equations [250] and nonlinear evolution equations in higher dimensions [251];
- Degasperis–Processi equation [252,253] and internal wave equation [254];
- differential–difference equations [255], etc. [256,257].

The obtained exact solutions have been used to describe various phenomena. Several examples are:

- the description of optical pulses [258,259,260,261,262,263,264,265], the propagation of optical solitons in fibers [266,267,268,269,270,271,272,273,274,275], and discrete solitons in optical systems [276,277];
- solitons in condensed matter physics [278]; solitons in lattices [279,280,281,282,283,284,285,286,287,288,289,290,291,292,293,294,295,296,297,298,299]; solitons in Heisenberg chains [300,301,302,303,304]; spin-wave solitons in magnetic films [305]; solitons in liquid crystals [306]; solitons in elastic solids [307]; solitons in rods, plates, and shells [308]; and collisions of solitons in spin systems [309];
- solitons in plasmas [310], for example, ion-acoustic solitons [311,312,313,314,315] and Langmuir solitons [316];
- solitons in fluid mechanics, for example, water waves [317,318,319,320,321,322,323,324]. This is a large area of research as the model equations used in fluid mechanics can often be solved analytically and the corresponding solutions describe solitons and multisolitons. Several examples are the observation of solitons in a sea [325], the description of internal waves in the atmosphere and atmospheric solitons [326,327,328], solitons in film flows [329], solitary waves and liquid drops [330], nonlinear soliton wave–beach interaction [331], long internal waves in deep fluid [332], solitons in rotating baroclinic fluid [333], ocean waves [334], solitary waves in two-fluid media [335], the propagation of bores [336,337], Peregrine soliton [338], solitary Rossby waves [339], and nonlinear focusing of waves in a fluid of finite depth [340];
- solitons in molecular structures [341,342,343] such as solitary waves in large blood vessels [344], dissipative solitons [345], and solitary excitations in muscle proteins [346];
- solitons connected to the Thirring model [347,348], the relationship between quantum mechanics and solitons on an elastic road [349], and the interpretation of solitons as particles and oscillators [350].

In addition, there is a large amount of literature on the various properties of solitons and processes with the participation of solitons. Several examples are breaking solitons [351], solitons and collapses [352]; solitons in non-uniform and disordered media [353,354]; bifurcations of solitons [355]; dynamics of solitons under perturbations [356,357,358]; vortex solitons [359], positons [360]; coupled solitons [361]; multidimensional solitons [362]; collisions of solitary waves [363]; the connection between strings, vortices, and solitons [364]; the interaction between short and long waves [365]; and wave–wave scattering [366].

The methodology of the Inverse Scattering Transform method stimulated the research and obtainment of many interesting mathematical results. Several examples are:

- Results on integrability: integrable Hamiltonian systems [367,368,369,370], asymptotic integrability [371,372], integrable nonlocal nonlinear equations [373], Lax pair for the one-dimensional Hubbard model [374] and higher-order models of water waves [375], the integrability of higher-dimensional discrete systems [376], geometrical aspects of solitons [377,378], conservation laws for nonlinear evolution equations [379], perturbed integrable equations [380], and the relationships between different methods for obtaining exact solutions of integrable systems [381];
- Stability of solitary waves [382,383,384];
- Pseudopotentials connected to nonlinear differential equations [385,386];
- Algebraic results and results connected to symmetries: the relationship between integrable systems and groups of Lee [387], the results from algebraic theory applicable to the theory of nonlinear equations [388], symmetries and the integrability of nonlinear differential equations [389,390,391], similarity reductions of the Boussinesq equation [392], symmetries, Lax pairs and solutions for AKNS systems [393,394,395], and Katz–Moodey–Lee algebras and solitons [396];
- Painlevé analysis of nonlinear differential equations [397,398,399,400,401], the connection between nonlinear evolution equations and ordinary differential equations of P-type [402,403], and perturbation theory for solitons [404];
- The results from rational solutions and other solutions of nonlinear evolution equations [405,406] and connections between the AKNS and KP hierarchies [407];
- The results from transformations of nonlinear differential equations: the direct linearization of nonlinear difference equations [408], gauge transformations applicable to solitons [409], Bäcklund transformations [410,411], the inverse spectral transform [412], generalized Fourier transforms for soliton equations [413], the singular manifold method for recovering Lax pairs and Darboux transformations [414], and the study of Bäcklund–Darboux transformations [415];
- The results from solutions of nonlinear differential equations: Wronskian representation of *N*-soliton solutions [416], asymptotic solutions of nonlinear evolution equations [417], and the dynamics of classical solitons in nonintegrable systems [418].

Numerical analysis of nonlinear waves is of special interest [419]. Several results in this research area are connected to methods for the symplectic integration of Hamiltonian systems [420], the finite difference and finite element methods for the Korteweg-de Vries equation [421,422,423,424], the Galerkin method [425,426,427,428,429], and the collocation and radial basis function methods for the Korteweg-de Vries equation [430]. The numerical methods have been successfully applied to, for example, the study of the evolution of solitary waves over a shelf [431], Lax pairs symbolic computations [432], the nonisospectral scattering problems [433], the numerical solution of the nonlinear Schrödinger equation [434], the numerical study of internal wave solitons [435], and the study of the soliton decay of nonlinear Alvfen waves [436].

This concludes the brief survey of the literature, noting that it should be considered an introduction to the vast area of research on nonlinear differential equations and the methods for obtaining exact analytical solutions of such equations. Next, we present a brief history of the research that led to the appearance of the methodology, which is the focus of this review article.

As we have seen, research on the methodology for obtaining exact solutions of nonlinear differential equations is important. In the early years of these studies, there was an attempt to remove the nonlinearity of the studied equation by an appropriate transformation. An example is the Hopf–Cole transformation [437,438]. This transformation converts the nonlinear Burgers equation to the linear heat equation. Another transformation connected the nonlinear Korteweg-de Vries equation to the linear Schrödinger equation. Efforts to obtain such transformations led to the method of the Inverse Scattering Transform [439], as well as the method of Hirota [136,141]. The truncated Painlevé expansions also supplied suitable transformations of the nonlinearities [440,441,442,443]. The possibilities connected to the Painlevé expansions were also explored by Kydryashov [444], who used a truncated Painlevé expansion. In these expansions, the truncation occurs after the constant whose term is kept in the expansion. Kudryashov [445] formulated the Method of the Simplest Equation (MSE). The method is based on the determination of the singularity order *n* of the solved NPDE. Then, a particular solution of this equation is searched as a power series of solutions of a simpler equation called the simplest equation. For further results on this methodology and its applications, see [446,447,448,449,450,451,452,453,454,455,456,457,458,459].

About 13 years ago we started the development of a methodology for obtaining exact and approximate solutions of nonlinear differential equations. This methodology is now called the SEsM (Simple Equations Method) [460,461,462,463,464,465,466,467,468,469,470,471]. Elements of this methodology were used in our articles written decades ago [472,473,474,475]. In 2009 and 2010 [476,477], we used the ODE of Bernoulli as the simplest equation [478] in the first version of the method, the Modified Method of the Simplest Equation (MMSE), and applied the methodology to population dynamics and ecology [479]. The MMSE [480,481] uses the concept of balance equations for the fixation of the simplest equation. After this, the searched solution is constructed as a truncated power series of the solution of the simplest equation. This methodology led to equivalent results with respect to the Method of the Simplest Equation mentioned above.

The MMSE was actively used by us until 2018 [482,483,484,485,486,487]. A special role in this period was played by article [486]. There, the methodology of the MMSE was extended by the use of a class of equations ${\left(\frac{d^{k}g}{d\xi^{k}}\right)}^{l}=\sum_{j=0}^{m}d_{j}g^{j}$ as the simplest equations. Here, k=1,…, l=1,…, and *m* and $d_{j}$ are parameters. The solution of this equation contains specific cases of many well-known functions such as hyperbolic functions, trigonometric functions, the elliptic functions of Weierstrass and Jacobi, etc. The capacity of the methodology has been extended over the years. The current version of the methodology (SEsM) can use more than one simple equation. The SEsM based on two simple equations was used in [488]. The first discussion of the SEsM was in [460]. Further discussions on the SEsM can be seen in [461,462,463,464,465]. Applications of specific cases of the SEsM can be seen in [489,490,491,492,493,494,495].

Below, we discuss the SEsM in detail. The discussion covers the use of transformations to convert the nonlinearity of the solved equation to a more treatable kind of nonlinearity, the use of composite functions in the SEsM and the application of the Faa di Bruno formula for derivatives of these functions, the role of simple equations and a special class of simple equations that define an interesting special function, as well as a series of polynomials, which appear frequently in the application of the SEsM.

The text is organized as follows. In Section 2, the methodology of the SEsM is described. In Section 3, we discuss the use of the transformations of the nonlinearity of the solved equation using the SEsM. These transformations convert various kinds of nonlinearities to a polynomial kind of nonlinearity which is more easily treatable by the SEsM. In Section 4, the use of the composite functions of the SEsM is discussed with examples of such use. In Section 5, the role of simple equations in the SEsM is described. In Section 6, the connection of the SEsM to the inverse scattering transform method and the method of Hirota is discussed. Section 7 is devoted to the specific application of the SEsM for obtaining the exact solution of a differential equation connected to the SIR model of epidemic spreading, with a possible application for analysis of epidemic waves of a class of epidemics, including COVID-19. In Section 8, additional applications of the SEsM are presented. In Section 9, we discuss the conjecture about the connection of the SEsM with other methods for obtaining specific solutions on nonlinear nonintegrable differential equations. Several concluding remarks are summarized in Section 10.

## 2. The Simple Equations Method (SEsM)

The SEsM has the goal of obtaining exact and approximate solutions of nonlinear differential equations. The algorithm of the SEsM is designed for solving systems of *n* nonlinear differential equations. The solution is built by known exact solutions of *m* simpler differential equations [470]. Here, we describe a specific case of the SEsM for the solution of a single nonlinear differential equation based on the solution of *m* simpler equations. The MMSE is the specific case. There, m=1.

The discussed version of the SEsM has four steps. The solved differential equation is

(1) $$A[u_{1}\left(x,\ldots ,t\right),\ldots ,u_{n}\left(x,\ldots ,t\right)]=0.$$

$A[u_{1}\left(x,\ldots ,t\right),\ldots ,u_{n}\left(x,\ldots ,t\right),\ldots ]$ depends on the functions $u_{1}\left(x,\ldots ,t\right),\ldots ,u_{n}\left(x,\ldots ,t\right)$ and some of their derivatives. The functions $u_{i}$ can depend on several spatial coordinates. The idea of the SEsM is to transform (1) to

(2) $$\sum r_{i}\left(\ldots \right)B_{i}=0.$$

Here, $B_{i}$ are some functions. $r_{i}$ are the relationships among the parameters of the solved equation and the parameters of the solution. We set

(3) $$r_{i}=0.$$

Thus, a system of nonlinear algebraic equations is obtained. Each nontrivial solution of (3) leads to a solution of the solved differential equation.

The steps for the transition from (1) to (3) are as follows. First, the transformation

(4) u(x,...,t)=T[F(x,…,t),G(x,…,t),…],

is applied. T(F,G,…) is a composite function of the other functions F,G,…. These functions can be the functions of several spatial variables and time. If possible, the transformation can remove the nonlinearity of the solved equation. An example is the Hopf–Cole transformation, which reduces the nonlinear Burgers equation to the linear heat equation [437,438]. The removal of the nonlinearity is rarely achieved. In most cases, the transformation *T* transforms the nonlinearity of the solved equation to an easily treatable kind of nonlinearity such as polynomial nonlinearity. Examples of the transformation *T* are $u\left(x,t\right)=4\tan^{-1}\left[F\left(x,t\right)\right]$ for the sine-Gordon equation and $u\left(x,t\right)=4\tanh^{-1}\left[F\left(x,t\right)\right]$ for the Poisson–Boltzmann equation [472,473,474,475]. The Painlevé expansion can be another appropriate transformation. u(x,...,t)=F(x,...,t) (no transformation) is also a possibility for certain classes of nonlinear differential equations. The application of (4) to (1) leads to a treatable nonlinear differential equation for $F_{i},G_{i},\ldots$. The transformation *T* may remain unfixed in the first step of the SEsM. Thus, the function *T* will be unknown and we have to determine it in some of the other three steps of the methodology.

Step 2 of the SEsM deals with the selection of the functions F(x,...,t), G(x,…,t), …. They are the composite functions of the known solutions of simpler differential equations. We can let the form of the composite functions be undetermined. This, together with the form of the transformation *T*, can be determined in Step 3 of the SEsM. Often, *F*, *G*, …, are fixed in Step 2 of the SEsM. A form that is often used is

(5) $$\begin{array}{c} F=\alpha +\sum_{i_{1}=1}^{N}\beta_{i_{1}}f_{i_{1}}+\sum_{i_{1}=1}^{N}\sum_{i_{2}=1}^{N}\gamma_{i_{1},i_{2}}f_{i_{1}}f_{i_{2}}+\sum_{i_{1}=1}^{N}\cdots \sum_{i_{N}=1}^{N}\sigma_{i_{1},\ldots ,i_{N}}f_{i_{1}}\ldots f_{i_{N}}. \end{array}$$

$\alpha ,\beta_{i_{1}},\gamma_{i_{1},i_{2}},\sigma_{i_{1},\ldots ,i_{N}}\ldots$ are parameters. The relationship used by Hirota [141] is a specific case of (5). Another specific case of (5) is the power series $F=\sum_{i=0}^{N}\mu_{n}f^{n}$ (μ is a parameter). This is frequently used in the MMSE.

Step 3 of the SEsM is often the most important in the application of the methodology. Here, one can determine the forms of the more simple equations and the composite functions *T*, *F*, *G*, …. All these are determined from the requirements for the satisfaction of the balance equations. The balance equations occur due to the requirement that any of $r_{i}$ from (3) must have at least two terms in order to ensure that a nontrivial solution of the solved equation is obtained. This determines the shapes of all composite functions and the forms of all used simpler equations.

The satisfaction of the balance equations in Step 3 leads to a system of nonlinear algebraic equations (3). This system contains relationships between the parameters of the solved equation and the parameters of the solution. In Step 4 of the SEsM, one solves the system (3). Each nontrivial solution of (3) leads to a nontrivial solution of the solved Equation (1).

## 3. The SEsM and the Transformation of the Nonlinearity of the Solved Equation

Step 1 of the SEsM is connected to the transformation of the nonlinearity of the solved equation (if necessary). If the nonlinearity of the solved equation is a polynomial one, there is no need for a transformation. Steps 2–4 of the SEsM can be applied directly in this case. In the case of the solved equation, which contains nonpolynomial nonlinearity, in Step 1 of the SEsM, one can attempt the application of a transformation that can transform the nonpolynomial nonlinearity to a polynomial nonlinearity. The following proposition can be performed, which was proved in [469].

**Proposition** **1.** *Let us consider a differential equation for the function u(x,…,t), which contains two kinds of terms:*

*1.*  *Terms containing only derivatives of u;*
*2.*  *Terms containing one or several nonpolynomial nonlinearities of the function u and these nonpolynomial nonlinearities are of the same kind.*

*Let u=T(F) be a transformation with the following properties:*

*1.*  *Property 1: The transformation T transforms any of the nonpolynomial nonlinearities to a function that contains only polynomials of F.*
*2.*  *Property 2: The transformation T transforms the derivatives of u to terms containing only the polynomials of the derivatives of F or the polynomials of the derivatives of F multiplied or divided by the polynomials of F.*

*Then, the transformation T transforms the studied differential equation into a differential equation containing only the polynomial nonlinearity of F.*

The above proposition allows us to transform numerous nonpolynomial nonlinearities into polynomial nonlinearities. A list of some nonpolynomial nonlinearities that can be transformed into polynomial nonlinearities can be found in [469]

Case 1: N(u)=exp(u); $N\left(u\right)={\left[\exp\left(u\right)\right]}^{m}$. The transformation is u=ln(F).
Case 2: N(u)=sin(u); $N\left(u\right)={\left[\sin\left(u\right)\right]}^{m}$. A possible transformation is $u=4\tan^{-1}\left(F\right)$.
Case 3: N(u)=cos(u); $N\left(u\right)={\left[\cos\left(u\right)\right]}^{m}$. The transformation is $u=4\tan^{-1}\left(F\right)$.
Case 4: N(u)=tan(u); $N\left(u\right)={\left[\tan\left(u\right)\right]}^{m}$. A possible transformation is $u=\tan^{-1}\left(F\right)$.
Case 5: N(u)=cot(u); $N\left(u\right)={\left[\cot\left(u\right)\right]}^{m}$. The transformation in this case is $u=\cot^{-1}\left(F\right)$.
Case 6: N(u)=sinh(u); $N\left(u\right)={\left[\sinh\left(u\right)\right]}^{m}$. The transformation is $u=4\tanh^{-1}\left(F\right)$.
Case 7: N(u)=cosh(u); $N\left(u\right)={\left[\cosh\left(u\right)\right]}^{m}$. The transformation is $u=4\tanh^{-1}\left(F\right)$.
Case 8: N(u)=tanh(u); $N\left(u\right)={\left[\tanh\left(u\right)\right]}^{m}$. The transformation is $u\left(F\right)=\tanh^{-1}\left(F\right)$. N(u).
Case 9: N(u)=coth(u); $N\left(u\right)={\left[\coth\left(u\right)\right]}^{m}$. The transformation is $u\left(F\right)=\coth^{-1}\left(F\right)$.
Case 10: N(u)=sin(mu); N(u)=cos(mu). The transformation is $u=4\tan^{-1}F$.

One illustrative example of the application of a transformation in Step 1 of the SEsM is for the equation

(6) $$bu_{xx}^{2}+du_{tt}^{2}=l\sin^{2}\left(u\right).$$

*b*, *d*, and *l* are parameters. In Step 1 of the SEsM, we use the transformation $u=4\tan^{-1}\left(F\right)$. The result is the conversion of (6) to an equation that contains only polynomial nonlinearities:

(7) $$\begin{array}{c} 4F^{2}\left(bF_{x}^{4}+dF_{t}^{4}\right)-4\left(F+F^{3}\right)\left(bF_{x}^{2}F_{xx}+dF_{t}^{2}F_{tt}\right)+\left(1+2F^{2}\right)\left(bF_{xx}^{2}+dF_{tt}^{2}\right)+F^{4}\left(bF_{xx}+dF_{tt}\right)-\\ l(F^{6}-2F^{4}+F^{2})=0. \end{array}$$

Step 2 of the SEsM is connected with the choice of *F* as a composite function of simpler functions. In our case, the choice is a specific case of the composite function $F\left(x,t\right)=F[T_{1}\left(x,t\right),T_{2}\left(x,t\right)]$, namely $F\left(x,t\right)=AT_{1}\left(\mu \right)T_{2}\left(\xi \right)$. Here, μ=αx, ξ=γt.

In Step 3 of the SEsM, one has to determine the form of the functions $T_{1}$ and $T_{2}$ in such a way that the balance equation leads to a balanced system of nonlinear algebraic relationships. The choice is

(8) $$\begin{array}{c} {T_{1}}_{\mu }^{2}=\sum_{i=0}^{N_{1}}\delta_{i}T_{1}^{i};\;\;\;{T_{2}}_{\xi }^{2}=\sum_{j=0}^{N_{2}}\epsilon_{i}T_{2}^{j}. \end{array}$$

Above, $\delta_{i}$ and $\epsilon_{i}$ are parameters. The balance equation has a specific solution $N_{1}=N_{2}=4$. Then, ${T_{1}}_{\mu }^{2}=\delta_{4}T_{1}^{4}+\delta_{3}T_{1}^{3}+\delta_{2}T_{1}^{2}+\delta_{1}T_{1}+\delta_{0};\;\;\;{T_{2}}_{\xi }^{2}=\epsilon_{4}T_{2}^{4}+\epsilon_{3}T_{2}^{3}+\epsilon_{2}T_{2}^{2}+\epsilon_{1}T_{1}+\epsilon_{0}.$ For simplicity, specific cases of the above simple equations are considered, namely

(9) $$\begin{array}{c} {T_{1}}_{\mu }^{2}=pT_{1}^{4}+qT_{1}^{2}+r;\;\;\;{T_{2}}_{\xi }^{2}=sT_{2}^{4}+vT_{2}^{2}+w. \end{array}$$

The form of the solution and the choice of the simple equations lead to the following balance system of nonlinear relationships in Step 4 of the SEsM:

(10) $$\begin{array}{ccc} b\alpha^{4}q^{2}+d\gamma^{4}v^{2} & = & l,\\ d\gamma^{4}vA^{2}w-b\alpha^{4}pq & = & 0,\\ -4d\gamma^{4}sw-b\alpha^{4}q^{2}-4b\alpha^{4}pr+l-d\gamma^{4}v^{2} & = & 0,\\ b\alpha^{4}A^{4}r^{2}+d\gamma^{4}s^{2} & = & 0,\\ -b\alpha^{4}qA^{2}r+d\gamma^{4}sv & = & 0,\\ b\alpha^{4}p^{2}+d\gamma^{4}A^{4}w^{2} & = & 0,\\ -d\gamma^{4}sv+b\alpha^{4}qA^{2}r & = & 0,\\ -d\gamma^{4}vA^{2}w+b\alpha^{4}pq & = & 0. \end{array}$$

A nontrivial solution of this system is

(11) $$p=w=r=s=0,\;\;q=\delta \frac{{\left[-b\left(d\gamma^{2}v^{2}-l\right)\right]}^{1/2}}{\alpha^{2}b},\;\;\delta =\pm 1,\;\;-b\left(d\gamma^{2}v^{2}-l\right)\geq 0.$$

*v*, *A*, *l*, *b*, *d*, α, γ are free parameters that satisfy the condition $-b(d\gamma^{2}v^{2}-l)\geq 0$. The corresponding solution of (6) is

(12) $$u\left(x,t\right)=4\tan^{-1}\left\{A\exp\left[\delta_{1}\left(\alpha vx+\gamma \delta \frac{{\left[-b\left(d\gamma^{2}v^{2}-l\right)\right]}^{1/2}}{\alpha^{2}b}t\right)\right]\right\},\;\;\delta_{1}=\pm 1.$$

We note that in (12), one can group the free parameters. These groups are *A*, αv, and $\gamma \delta \frac{{\left[-b\left(d\gamma^{2}v^{2}-l\right)\right]}^{1/2}}{\alpha^{2}b}$. Thus, the free parameters for Solution (12) are reduced to three. Let us obtain this result by obtaining (12) in another way. Above, we used a composite function of the kind $F\left(x,t\right)=AT_{1}\left(\mu \right)T_{2}\left(\xi \right)$; μ=αx, ξ=γt, in other words, we searched for standing waves. We can obtain (12) based on a search for the traveling wave solutions of (6), where u(x,t)=u(z)=u(x−ct). *c* is the velocity of the traveling wave. We introduce the parameter $a^{2}=l/\left(b+dc^{2}\right)$ and the velocity becomes

(13) $$c=\frac{l-ba^{2}}{da^{2}}.$$

Then, (6) can be factorized: $u_{zz}^{2}-a^{2}\sin^{2}\left(u\right)=\left(u_{zz}+\sin\left(u\right)\right)\left(u_{zz}-\sin_{u}\right)=0$, and this leads to the following solution of (6):

(14) $$u=4\tan^{-1}\left\{\frac{1+k\mathrm{sn}\left[a^{1/2}\left(x-\sqrt{\frac{l-ba^{2}}{da^{2}}t}+z_{0}\right),k\right]}{\mathrm{dn}\left[a^{1/2}\left(x-\sqrt{\frac{l-ba^{2}}{da^{2}}t}+z_{0}\right),k\right]}\right\}.$$

In (14), sn and dn are the Jacobi elliptic functions of modulus *k*, and $z_{0}$ is a constant of integration. Now, let k=1. The Jacobi elliptic functions reduce to hyperbolic functions and (14) is reduced to its specific case

(15) $$u=4\tan^{-1}\left\{2\exp\left[a^{1/2}\left(x-\sqrt{\frac{l-ba^{2}}{da^{2}}t}+z_{0}\right)\right]\right\}.$$

Here, we have *a* and $A=2\exp(a^{1/2}z_{0})$ as free parameters. However, we have another free parameter and it is connected to the group of parameters $\frac{l-ba^{2}}{da^{2}}$. For this group of parameters, we have the relationship (13). Let us fix *a* and $z_{0}$. This does not fix *c* in (13) as *l*, *b*, and *d* are not fixed. This (15) has effectively three free parameters, similar to (12). We are grateful to the anonymous reviewer who suggested discussing the number of free parameters for the solution (12) using the considerations described in (13)–(15).

## 4. Composite Functions and Their Role in the Algorithm of SEsM

One encounters composite functions in several places of the algorithm of the SEsM. The presence of composite functions leads to preferences in the choice of the simple equations and the occurrence of specific sets of polynomials in the SEsM. Below, we discuss this in detail.

The differential equations contain derivatives of unknown functions. If the unknown functions are composite, then one has to use the Faa di Bruno formula for the derivative of a composite function. The Faa di Bruno formula is written below for the function $h(x_{1},\ldots ,x_{d})$ of *d* independent variables $x_{1},\ldots ,x_{d}$, which is a composite function of *m* other functions $g_{1}^{\left(1\right)},\ldots ,g^{\left(m\right)}$

(16) $$h\left(x_{1},\ldots ,x_{d}\right)=f\left[g^{\left(1\right)}\left(x_{1},\ldots ,x_{d}\right),\ldots ,g^{\left(m\right)}\left(x_{1},\ldots ,x_{d}\right)\right].$$

The following notations are used:

1. $\vec{\nu }=\left(\nu_{1},\ldots ,\nu_{d}\right)$: *d*-dimensional index containing integer non-negative numbers $\nu_{1},\ldots ,\nu_{d}$.
2. $\vec{z}=\left(z_{1},\ldots ,z_{d}\right)$: *d*-dimensional object containing real numbers $z_{1},\ldots ,z_{d}$.
3. $|\vec{\nu }|=\sum_{i=1}^{d}\nu_{i}$: sum of the elements of the *d*-dimensional index $\vec{\nu }$.
4. $\vec{\nu }!=\prod_{i=1}^{d}\nu_{i}!$: factorial of the multicomponent index $\vec{\nu }$.
5. ${\vec{z}}^{\vec{\nu }}=\prod_{i=1}^{d}z_{i}^{\nu_{i}}$: $\vec{\nu }$-th power of the multicomponent variable $\vec{z}$.
6. $D_{\vec{x}}^{\vec{\nu }}=\frac{\partial^{|\vec{\nu }|}}{\partial x_{1}^{\nu_{1}}\ldots \partial x_{d}^{\nu_{d}}}$, $|\vec{\nu }|>0$: $\vec{\nu }$-th derivative with respect to the multicomponent variable $\vec{x}$. Then, the identity operator is denoted as $D_{\vec{x}}^{\vec{0}}$.
7. $\|\vec{z}\|=\max|z_{i}|$: maximum value component of the multicomponent variable $\vec{z}$ for the interval 1≤i≤d.
8. For the *d*-dimensional index $\vec{l}=\left(l_{1},\ldots ,l_{d}\right)$ ($l_{1},\ldots ,l_{d}$ are integers), it follows that $\vec{l}\leq \vec{\nu }$ when $l_{i}\leq \nu_{i},i=1,\ldots ,d$. The following notation is used:
  ν→l→=∏i=1dνili=ν→!l→!(ν→−l→)!.
9. Ordering of vector indexes: for two vector indexes $\vec{\mu }=\left(\mu_{1},\ldots ,\mu_{d}\right)$ and $\vec{\nu }=\left(\nu_{1},\ldots ,\nu_{d}\right)$, we have $\vec{\mu }≺\vec{\nu }$ when one of the following holds:
  (a) $|\vec{\mu }|<|\vec{\nu }|$.
  (b) $|\vec{\mu }|=|\vec{\nu }|$ and $\mu_{1}<\nu_{1}$.
  (c) $|\vec{\mu }|=|\vec{\nu }|$, $\mu_{1}=\nu_{1}$, … $\mu_{k}=\nu_{k}$ and $\mu_{k+1}<\nu_{k+1}$ for some 1≤k<d.

In addition, the following notation is used:

(17) $$h_{\left(\vec{\nu }\right)}=D_{\vec{x}}^{\vec{\nu }}h;\;\;\;f_{\left(\vec{\lambda }\right)}=D_{\vec{y}}^{\vec{\lambda }}f;\;\;\;g_{\left(\vec{\mu }\right)}^{\left(i\right)}=D_{\vec{x}}^{\vec{\mu }}g^{\left(i\right)};\;\;\;{\vec{g}}_{\left(\vec{\mu }\right)}=\left(g_{\left(\vec{\mu }\right)}^{\left(1\right)},\ldots ,g_{\left(\vec{\mu }\right)}^{\left(m\right)}\right).$$

Based on the above notation, the Faa di Bruno for the composite derivative of a function containing the functions of many variables is [496]

(18) $$h_{\left(\vec{\nu }\right)}=\sum_{1\leq |\vec{\lambda }|\leq n}f_{\left(\vec{\lambda }\right)}\sum_{s=1}^{n}\sum_{p_{s}\left(\vec{\nu },\vec{\lambda }\right)}\left(\vec{\nu }!\right)\prod_{j=1}^{s}\frac{{\left[{\vec{g}}_{\left({\vec{l}}_{j}\right)}\right]}^{{\vec{k}}_{j}}}{\left({\vec{k}}_{j}!\right){\left[{\vec{l}}_{j}!\right]}^{|{\vec{k}}_{j}|}}.$$

In (18) $n=|\vec{\nu }|$ and

(19) $$p_{s}\left(\vec{\nu },\vec{\lambda }\right)=\left\{{\vec{k}}_{1},\ldots ,{\vec{k}}_{s};{\vec{l}}_{1},\ldots ,{\vec{l}}_{s}\right\},\;\;\;|{\vec{k}}_{i}|>0;\;\;\;\;\;0≺{\vec{l}}_{1}\ldots ≺{\vec{l}}_{s},\;\;\;\sum_{i=1}^{s}{\vec{k}}_{i}=\vec{\lambda },\;\;\;\sum_{i=1}^{s}|{\vec{k}}_{i}|{\vec{l}}_{i}=\vec{\nu }.$$

For example, the derivatives of the composite function of two independent variables $x_{1}$ and $x_{2}$$h\left(x_{1},x_{2}\right)=f\left[g^{\left(1\right)}\left(x_{1},x_{2}\right),\ldots ,g^{\left(m\right)}\left(x_{1},x_{2}\right)\right]$ are

(20) $$\begin{array}{c} h_{\left(\vec{\nu }\right)}=\frac{\partial^{\nu_{1}+\nu_{2}}h}{\partial x_{1}^{\nu_{1}}\partial x_{2}^{\nu_{2}}}=\sum_{1\leq \left(\lambda_{1}+\ldots +\lambda_{m}\right)\leq \nu_{1}+\nu_{2}}\frac{\partial^{\lambda_{1}+\ldots +\lambda_{m}}f}{\partial {g^{\left(1\right)}}^{\lambda_{1}}\ldots \partial {g^{\left(m\right)}}^{\lambda_{m}}}\{\sum_{s=1}^{\nu_{1}+\nu_{2}}\sum_{p_{s}\left(\vec{\nu },\vec{\lambda }\right)}\left(\nu_{1}!\nu_{2}!\right)\times \\ \prod_{j=1}^{s}\left[\frac{1}{\left(k_{j,1}!\ldots k_{j,m}!\right){\left(l_{j,1}!+l_{j,2}!\right)}^{k_{j,1}+\ldots +k_{j,m}}}\prod_{i=1}^{m}{\left(\frac{\partial^{l_{j,1}+l_{j,2}}}{\partial x_{1}^{l_{j,1}}\partial x_{2}^{l_{j,2}}}g^{\left(i\right)}\right)}^{k_{j,i}}\right]\}. \end{array}$$

The specific case of a composite function containing one function of one variable h=f[g(x)] is of special interest, as it is often used in practice. For this case, the Faa di Bruno formula is

(21) $$h_{\left(n\right)}=\sum_{k=1}^{n}f_{\left(k\right)}\sum_{p(k,n)}n!\prod_{i=1}^{n}\frac{g_{\left(i\right)}^{\lambda_{i}}}{\left(\lambda_{i}!\right){\left(i!\right)}^{\lambda_{i}}}.$$

In (21), $h_{\left(n\right)}=\frac{d^{n}h}{dx^{n}}$; $f_{\left(k\right)}=\frac{d^{k}f}{dg^{k}}$; and $g_{\left(i\right)}=\frac{d^{i}g}{dx^{i}}$. In addition, $p\left(n,k\right)=\left\{\lambda_{1},\lambda_{2},\ldots ,\lambda_{n}\right\}$ is a set of numbers satisfying $\sum_{i=1}^{n}\lambda_{i}=k;\sum_{i=1}^{n}i\lambda_{i}=n.$

Two results of the composite functions are of interest to the methodology of the SEsM. The first is connected to the use of simple equations for exponential functions [470]

**Theorem** **1.** *Let us consider a nonlinear partial differential equation that contains a polynomial P of the function $h(x_{1},x_{2})$ and its derivatives. The relationship for this equation is*

(22) $$P(h,h_{{\vec{\nu }}_{1}},\ldots ,h_{{\vec{\nu }}_{N}})=0.$$

*Above, N can be any natural number. We search for the solution of the above equation in the form*

$$h\left(x_{1},x_{2}\right)=f\left[g^{\left(1\right)}\left(x_{1},x_{2}\right),\ldots ,g^{\left(m\right)}\left(x_{1},x_{2}\right)\right],$$

*where h is a polynomial of the functions $g^{\left(1\right)}\left(x_{1},x_{2}\right),\ldots ,g^{\left(m\right)}\left(x_{1},x_{2}\right)$. Let each function $g^{\left(i\right)}\left(x_{1},x_{2}\right)$ satisfy the simple equation*

(23) $$g_{\left(x_{j}\right)}^{\left(i\right)}=\alpha_{i,j}g^{\left(i\right)},$$

*where $\alpha_{i,j}$ is a constant parameter. Then, the solved nonlinear PDE is reduced to a polynomial of the functions $g^{\left(1\right)}\left(x_{1},x_{2}\right),\ldots ,g^{\left(m\right)}\left(x_{1},x_{2}\right)$ containing monomials multiplied by some coefficients. These coefficients are nonlinear algebraic relations between the parameters of the solved equation and the parameters $\alpha_{i,j}$. We set the above coefficients to 0. The result is a system of nonlinear algebraic equations. Any nontrivial solution of this system leads to a solution of the solved nonlinear PDE.*

The second result is connected to the use of the composite function of a function of a single variable. In this case, one can use a more complicated simple equation in the SEsM. In more detail, we consider a nonlinear partial differential equation with nonlinearities that are polynomials of the unknown function h(x,t) and its derivatives. We search for a solution of the kind

(24) h(x,t)=h(ξ);ξ=μx+νt,

where μ and ν are parameters and *H* is a composite function of another function *g*:

(25) h=f[g(ξ)].

The assumption is that *f* is a polynomial of *g*:

(26) $$f=\sum_{r=0}^{q}b_{r}g^{r},$$

and the general form of the simple equation is

(27) $$g_{\left(k\right)}^{l}={\left(\frac{d^{k}g}{d\xi^{k}}\right)}^{l}=\sum_{j=0}^{m}a_{j}g^{j}.$$

Equation (27) defines the function $V_{a_{0},a_{1},\ldots ,a_{m}}(\xi ;k,l,m$). In this notation, *k* is the order of derivative of *g*, *l* is the degree of the derivative, and *m* is the highest degree of the polynomial of *g* in the defining ODE. *V* has as specific cases the trigonometric, hyperbolic, and elliptic functions of Weierstrass and Jacobi, etc. The result we describe is connected to a specific case of the equation for the *V*-function $V_{a_{0},a_{1},\ldots ,a_{m}}\left(\xi ;1,2,m\right)$:

(28) $$g_{\left(1\right)}^{2}={\left(\frac{dg}{d\xi }\right)}^{2}=\sum_{j=0}^{m}a_{j}g^{j}.$$

The result is [486].

**Theorem** **2.** *If $g_{\left(1\right)}^{2}$ is given by Equation (28) and f is a polynomial of g given by Equation (26), then for h[f(g)], the following relationship holds:*

$$h_{\left(n\right)}=K_{n}\left(q,m\right)\left(g\right)+g_{\left(1\right)}Z_{n}\left(q,m\right)\left(g\right)$$

*where $K_{n}\left(q,m\right)\left(g\right)$ and $Z_{n}\left(q,m\right)\left(g\right)$ are polynomials of the function g(ξ).*

Note that for some values of *n*, one of the polynomials $K_{n}\left(q,m\right)$ or $Z_{n}\left(q,m\right)$ can be equal to 0.

The theorem states that the derivatives of the composite function *h* are constructed by specific polynomials. These polynomials are calculated by the recurrence relationships [486]

(29) $$\begin{array}{c} K_{n+1}=\frac{Z_{n}}{2}\sum_{j=0}^{m}ja_{j}g^{j-1}+\frac{dZ_{n}}{dg}\sum_{j=0}^{m}a_{j}g^{j};\;\;\;\;\;Z_{n+1}=\frac{dK_{n}}{dg}, \end{array}$$

with $K_{0}=\sum_{r=0}^{q}b_{r}g^{r}$ and $Z_{0}=0$. In this way, $K_{1}=0;\;\;Z_{1}=\sum_{r=0}^{q}rb_{r}g^{r-1},$ and, for example,

(30) $$\begin{array}{ccc} K_{4} & = & \sum_{r=0}^{q}\sum_{j=0}^{m}\sum_{u=0}^{m}\left[\left(\frac{1}{2}jr+r\left(r-1\right)\right)\left(j+r-2\right)\left(\frac{1}{2}u+j+r-3\right)\right]a_{j}b_{r}a_{u}g^{j+r+u-4},\\ Z_{4} & = & 0. \end{array}$$

Another kind of simple equation leads to another set of polynomials. The simple equation (*n* and $c_{j}$ are constant parameters)

(31) $$g_{\left(1\right)}=\sum_{j=0}^{n}c_{j}g^{j},$$

is frequently used. In this case [470], one obtains the following set of polynomials. One starts from $L_{0}=\sum_{r=0}^{q}b_{r}q^{r}$ and uses the recurrence relationship

(32) $$L_{i+1}=\frac{dL_{i}}{dg}\sum_{j=0}^{m}c_{j}g^{j}.$$

The derivative of the composite function is $h_{\left(n\right)}=L_{n}\left(g\right)$, for example,

(33) $$\begin{array}{c} L_{3}=\sum_{r=0}^{q}\sum_{j=0}^{m}\sum_{k=0}^{m}\sum_{l=0}^{m}b_{r}r\left(r+j-1\right)\left(r+j+k-2\right)c_{j}c_{k}c_{l}g^{r+j+k+l-3}. \end{array}$$

Let us consider two examples of the application of the SEsM with the participation of composite functions. The first example is for the generalized Korteweg-de Vries equation

(34) $$\frac{\partial u}{\partial t}+Au^{p}\frac{\partial u}{\partial x}+\frac{\partial^{3}u}{\partial x^{3}}=0,$$

In (34), *p* is a positive integer number and *A* is a parameter. The solution of Equation (34) is searched as a composite function u=h[f(g(ξ))], where ξ=αx+βt, *g* is the solution of the simplest Equation (28) and *f* is given by Equation (26). The substitution of u=h[f(g(ξ))] in Equation (34) and the application of the SEsM leads to an equation of the kind

(35) $$W_{0}\left(g\right)+W_{1}\left(g\right)g_{\left(1\right)}=0.$$

with

(36) $$\begin{array}{ccc} W_{0}\left(g\right) & = & \nu K_{1}\left(g\right)+\mu AK_{0}{\left(g\right)}^{p}K_{1}\left(g\right)+\mu^{3}K_{3}\left(g\right),\\ W_{1}\left(g\right) & = & \nu Z_{1}\left(g\right)+\mu AK_{0}{\left(g\right)}^{p}Z_{1}\left(g\right)+\mu^{3}Z_{3}\left(g\right). \end{array}$$

$K_{1}=K_{3}=0$ and the first equation of (35) is satisfied. The second equation needs balance. It is m=2+pq. For the case q=1, m=2+p. Then, from Equations (26) and (28) one obtains $h=b_{0}+b_{1}g;\;\;\;g_{\left(1\right)}^{2}=\sum_{j=0}^{2+p}a_{j}g^{j}$. Thus, the second equation from (36) is reduced to the system of nonlinear algebraic relationships among the parameters of Equation (34) and the parameters of the solution (Step 4 of the SEsM):

(37) νb1δ0,k+pkμAb0p−kb1k+1+12μ3(k+1)(k+2)ak+2b1=0,k=0,…,p.

δ is the delta-symbol of Kronecker.

The solution of Equation (37) is obtained for $b_{0}=0$. Then, one obtains the system of algebraic equations:

(38) $$\begin{array}{ccc} k & = & 0:\;\;\;\nu +\mu^{3}a_{2}=0,\\ k & = & 1:\;\;\;a_{3}=0,\\ & & \ldots \ldots \\ k & = & p-1:\;\;\;a_{p+1}=0,\\ k & = & p:\;\;\;Ab_{1}^{p}+\frac{1}{2}\mu^{2}\left(p+1\right)\left(p+2\right)a_{p+2}=0. \end{array}$$

The solution is $a_{2}=-\frac{\nu }{\mu^{3}};\;\;\;a_{p+2}=-\frac{2Ab_{1}^{p}}{\mu^{2}\left(p+1\right)\left(p+2\right)}.$ This fixes the simple equation, Equation (28), as

(39) $$g_{\left(1\right)}^{2}=-\frac{\nu }{\mu^{3}A}g^{2}-\frac{2b_{1}^{p}}{\mu^{2}A\left(p+1\right)\left(p+2\right)}g^{p+2}.$$

The solution of (39) is $g\left(\xi \right)=\frac{\Omega }{\cosh^{\omega }\left(\xi \right)},$ with $\omega =\frac{2}{p};\;\;\mu^{2}=\frac{p^{2}A\Omega^{p}b_{1}^{p}}{2(p+1)(p+2)};\;\;\nu =-\frac{4\mu^{3}}{p^{2}}$. The solution of (34) becomes

(40) $$u\left(\xi \right)=\frac{\Omega b_{1}}{\cosh^{2/p}\left(\xi \right)};\xi =\mu x+\nu t.$$

For the particular case p=1, A=−6 (34) coincides with the classical Korteweg-de Vries equation. The values $b_{1}=1$ and Ω=−2 of the parameters lead to μ=1, ν=−4. Then, (40) reduces to the single soliton solution of the KdV equation $u\left(x,t\right)=-\frac{2}{\cosh^{2}\left(x-4t\right)}$ One can show that (40) is the solution of Equation (34) for an arbitrary real nonzero value of *p* (and not only for positive integer values of *p*) [486].

In a similar manner, one can obtain numerous solutions of the Olver equation [486]

(41) $$\frac{\partial u}{\partial t}+\frac{\partial u}{\partial x}+\alpha_{0}u\frac{\partial u}{\partial x}+\alpha_{1}\frac{\partial u}{\partial x}\frac{\partial^{2}u}{\partial x^{2}}+\alpha_{2}u\frac{\partial^{3}u}{\partial x^{3}}+\alpha_{3}u^{2}\frac{\partial u}{\partial x}+\alpha_{4}\frac{\partial^{3}u}{\partial x^{3}}+\alpha_{5}\frac{\partial^{5}u}{\partial x^{5}}=0.$$

## 5. The Role of the Simple Equations in the SEsM

In general, the form of the solution of the solved nonlinear differential equation is connected to the form of the solution of the used simple equations by means of the balance equations, which have to be satisfied in Step 3 of the SEsM. We are not obligated to choose the form of the simple equations before Step 3 of the SEsM. However, if we do, additional restrictions are imposed on the form of the searched solution of the solved equation [483]. Below, we discuss this in more detail.

We consider a simple equation of the kind

(42) $$Q\left(\Phi ,\frac{d\Phi }{d\xi },\frac{d^{2}\Phi }{d\xi^{2}},\ldots ,\frac{d^{n}\Phi }{d\xi^{n}}\right)=0.$$

Using the solution Φ of this simple equation, we build a function F=F(Φ), which has to solve the more complicated equation

(43) $$P\left(F,\frac{dF}{d\xi },\frac{d^{2}F}{d\xi^{2}},\ldots ,\frac{d^{n}F}{d\xi^{n}}\right)=0.$$

We can choose *Q* and *F*. Thus, we have a function and the question is as follows: What is the manifold of equations that have this function as a solution? Below, are the responses to this question for the case of simple equations of the kind

(44) $${\left(\frac{d\Phi }{d\xi }\right)}^{\epsilon }=\sum_{\alpha =0}^{\beta }\gamma_{\alpha }{\left[\Phi \left(\xi \right)\right]}^{\alpha },$$

where ϵ and β are positive integers and the function F(Φ) is a polynomial

(45) $$F\left(\Phi \right)=\sum_{\mu =0}^{\nu }\theta_{\mu }{\left[\Phi \left(\xi \right)\right]}^{\mu }.$$

For the construction of equations of the kind (43), one needs to know the derivatives of *F* with respect to ξ. Some derivatives are

(46) $$\frac{dF}{d\xi }=\frac{dF}{d\Phi }\frac{d\Phi }{d\xi }.$$

(47) $$\frac{d^{2}F}{d\xi^{2}}=\frac{d^{2}F}{d\Phi^{2}}{\left(\frac{d\Phi }{d\xi }\right)}^{2}+\frac{dF}{d\Phi }\frac{d^{2}\Phi }{d\xi^{2}}.$$

(48) $$\frac{d^{3}F}{d\xi^{3}}=\frac{d^{3}F}{d\Phi^{3}}{\left(\frac{d\Phi }{d\xi }\right)}^{3}+3\frac{d^{2}F}{d\Phi^{2}}\frac{d\Phi }{d\xi }\frac{d^{2}\Phi }{d\xi^{2}}+\frac{dF}{d\Phi }\frac{d^{3}\Phi }{d\xi^{3}}.$$

(49) $$\frac{d^{4}F}{d\xi^{4}}=\frac{d^{4}F}{d\Phi^{4}}{\left(\frac{d\Phi }{d\xi^{4}}\right)}^{4}+6\frac{d^{3}F}{d\Phi^{3}}{\left(\frac{d\Phi }{d\xi }\right)}^{2}\frac{d^{2}\Phi }{d\xi^{2}}+3\frac{d^{2}F}{d\Phi^{2}}{\left(\frac{d^{2}\Phi }{d\xi^{2}}\right)}^{2}+4\frac{d^{2}F}{d\Phi^{2}}\frac{d\Phi }{d\xi }\frac{d^{3}\Phi }{d\xi^{3}}+\frac{dF}{d\Phi }\frac{d^{4}\Phi }{d\xi^{4}}.$$

(50) $$\begin{array}{c} \frac{d^{5}F}{d\xi^{5}}=\frac{d^{5}F}{d\Phi^{5}}{\left(\frac{d\Phi }{d\xi^{4}}\right)}^{5}+10\frac{d^{4}F}{d\Phi^{4}}{\left(\frac{d\Phi }{d\xi }\right)}^{3}\frac{d^{3}\Phi }{d\xi^{2}}+15\frac{d^{3}F}{d\Phi^{3}}\frac{d\Phi }{d\xi }{\left(\frac{d^{2}\Phi }{d\xi^{2}}\right)}^{2}+\\ 10\frac{d^{3}F}{d\Phi^{3}}{\left(\frac{d\Phi }{d\xi }\right)}^{2}\frac{d^{3}\Phi }{d\xi^{3}}+10\frac{d^{2}F}{d\Phi^{2}}\frac{d^{2}\Phi }{d\xi^{2}}\frac{d^{3}\Phi }{d\xi^{3}}+5\frac{d^{2}F}{d\Phi^{2}}\frac{d\Phi }{d\xi }\frac{d^{4}\Phi }{d\xi^{4}}+\frac{dF}{d\Phi }\frac{d^{5}\Phi }{d\xi^{5}}. \end{array}$$

Derivatives (46)–(50) have different forms for different forms of (45) and different versions of the simple equation, Equation (44). This results in different hierarchies of nonlinear PDEs, which have as their solutions the corresponding form of F(Φ). For an illustration of this, we consider as a simple equation the extended tanh-equation

(51) $$\frac{d\Phi }{d\xi }=b^{2}-a^{2}\Phi^{2},$$

which has the solution

(52) $$\Phi \left(\xi \right)=\frac{b}{a}\tanh\left[a\;b\;\left(\xi +\xi_{0}\right)\right].$$

In (52), $a^{2}\Phi {\left(\xi \right)}^{2}<d^{2}$ and $\xi_{0}$ is a constant of integration. Equation (52) can be used for the construction of traveling wave solutions of numerous nonlinear PDEs. Solution (51) leads to specific forms of Derivatives (46)–(50). The simplest specific case of Equation (45) is $\frac{dF}{d\Phi }=p_{1}$. Then,

(53) $$F\left(\Phi \right)=p_{0}+p_{1}\Phi =p_{0}+\frac{p_{1}b}{a}\tanh\left[a\;b\;\left(\xi +\xi_{0}\right)\right].$$

One obtains Derivatives (46)–(50):

(54) $$\frac{dF}{d\xi }=-p_{1}a^{2}\Phi^{2}+p_{1}b^{2}.$$

(55) $$\frac{d^{2}F}{d\xi^{2}}=2p_{1}a^{4}\Phi^{3}-2p_{1}a^{2}b^{2}\Phi .$$

(56) $$\frac{d^{3}F}{d\xi^{3}}=-6p_{1}a^{6}\Phi^{4}+8p_{1}a^{4}b^{2}\Phi^{2}-2p_{1}a^{2}b^{4}.$$

(57) $$\frac{d^{4}F}{d\xi^{4}}=24p_{1}a^{8}\Phi^{5}-40p_{1}a^{6}b^{2}\Phi^{3}+16p_{1}a^{4}b^{4}\Phi .$$

(58) $$\frac{d^{5}F}{d\xi^{5}}=-120p_{1}a^{10}\Phi^{6}+240p_{1}a^{8}b^{2}\Phi^{4}-136p_{1}a^{6}b^{4}\Phi^{2}+16p_{1}a^{4}b^{6}.$$

The function (53) is a solution of numerous nonlinear PDEs. Let us demonstrate the construction of some of these equations. The first equation is

(59) $$q_{1}\frac{d^{2}F}{d\xi^{2}}+q_{2}F\frac{dF}{d\xi }+q_{3}F^{3}+q_{4}F^{2}+q_{5}F+q_{6}=0.$$

For a solution of the kind (53), the derivatives of *F* are given by Equations (54) and (55). The substitution of (53)–(55) in Equation (59) leads to the proof of the following proposition.

**Proposition** **2** ([483])**.** *Each solution of the system of nonlinear algebraic relationships*

(60) $$\begin{array}{ccc} 2q_{1}a^{4}p_{1}^{2}+q_{3}p_{1}^{3}-q_{2}p_{1}^{3}a^{2} & = & 0,\\ 2q_{1}a^{4}p_{1}p_{0}-3q_{2}p_{0}a^{2}p_{1}^{2}+3q_{3}p_{0}p_{1}^{2}+q_{4}p_{1}^{2} & = & 0,\\ q_{5}p_{1}-2q_{2}p_{0}^{2}a^{2}p_{1}+q_{2}p_{1}\left(b^{2}-a^{2}p_{0}^{2}\right)-2q_{1}a^{2}p_{1}^{2}b^{2}+3q_{3}p_{0}^{2}p_{1}+2q_{4}p_{0}p_{1} & = & 0,\\ q_{2}p_{0}\left(b^{2}-a^{2}p_{0}^{2}\right)+q_{6}+q_{5}p_{0}-2q_{1}a^{2}p_{0}p_{1}b^{2}+q_{3}p_{0}^{3}+q_{4}p_{0}^{2} & = & 0. \end{array}$$

*leads to an exact solution of the kind (53) of Equation (59).*

Let us set, for example, $q_{3}=q_{4}=q_{6}=0$. Then, we obtain the conditions of a function of the kind (53) to be the solution of the equation

(61) $$q_{1}\frac{d^{2}F}{d\xi^{2}}+q_{2}F\frac{dF}{d\xi }+q_{5}F=0.$$

For this, the system of the following nonlinear algebraic equations must be satisfied

(62) $$\begin{array}{ccc} -q_{2}p_{1}^{3}a^{2}+2q_{1}a^{4}p_{1}^{2} & = & 0,\\ -3q_{2}p_{0}a^{2}p_{1}^{2}+2q_{1}a^{4}p_{1}p_{0} & = & 0,\\ -2q_{2}p_{0}^{2}a^{2}p_{1}+q_{2}p_{1}\left(b^{2}-a^{2}p_{0}^{2}\right)-2q_{1}a^{2}p_{1}^{2}b^{2}+q_{5}p_{1} & = & 0,\\ q_{2}p_{0}\left(b^{2}-a^{2}p_{0}^{2}\right)-2q_{1}a^{2}p_{0}p_{1}b^{2}+q_{5}p_{0} & = & 0. \end{array}$$

Let us now set $q_{6}=0$. Thus, we search for a solution of

(63) $$q_{1}\frac{d^{2}F}{d\xi^{2}}+q_{2}F\frac{dF}{d\xi }+q_{3}F^{3}+q_{4}F^{2}+q_{5}F=0.$$

One solution of (60) is

(64) $$\begin{array}{c} p_{0}=\frac{q_{4}}{2(a^{2}q_{2}-q_{3})};\;p_{1}=\frac{2a^{4}q_{1}}{a^{2}q_{2}-q_{3}};\;b=\frac{1}{2}\sqrt{\frac{\left(q_{4}^{2}-4q_{3}q_{5}+4a^{2}q_{2}q_{5}\right)}{\left(q_{2}q_{3}-a^{2}q_{2}^{2}+4q_{1}^{2}a^{6}\right)}}. \end{array}$$

The substitution of (64) in (53) leads to the following solution of Equation (63)

(65) $$F\left(\xi \right)=\frac{q_{4}}{2(a^{2}q_{2}-q_{3})}+\left(\frac{a^{3}q_{1}}{a^{2}q_{2}-q_{3}}\sqrt{\frac{\left(q_{4}^{2}-4q_{3}q_{5}+4a^{2}q_{2}q_{5}\right)}{\left(q_{2}q_{3}-a^{2}q_{2}^{2}+4q_{1}^{2}a^{6}\right)}}\right)\tanh\left[\frac{a}{2}\sqrt{\frac{\left(q_{4}^{2}-4q_{3}q_{5}+4a^{2}q_{2}q_{5}\right)}{\left(q_{2}q_{3}-a^{2}q_{2}^{2}+4q_{1}^{2}a^{6}\right)}}\;\left(\xi +\xi_{0}\right)\right].$$

The solution (65) is an exact solution of each nonlinear partial differential equation that can be reduced to Equation (63).

One can consider more complicated differential equations such as

(66) $$q_{1}\frac{d^{2}F}{d\xi^{2}}+q_{2}F\frac{dF}{d\xi }+q_{3}\frac{dF}{d\xi }+q_{4}F^{3}+q_{5}F^{2}+q_{6}F+q_{7}=0,$$

and to search for conditions for the existence of a solution of the kind (53). One can proceed by using more complicated forms of F(Φ). One such form is $\frac{dF}{d\Phi }=p_{1}+2p_{2}\Phi$. Then,

(67) $$F=p_{0}+p_{1}\Phi +p_{2}\Phi^{2}.$$

Φ(ξ) is (52). The substitution of (52) and (67) in (66) leads to a system of seven nonlinear algebraic equations that have to be solved in order to construct a solution of the kind (67) and (52) of (66). A nontrivial solution of this algebraic system is

(68) $$\begin{array}{c} a=\sqrt{\frac{q_{4}}{q_{2}}};\;q_{1}=0;\;q_{3}=\frac{q_{5}q_{2}}{q_{4}};q_{6}=\frac{q_{4}q_{7}}{q_{5}};\;b=\sqrt{-\frac{q_{4}q_{7}}{q_{2}q_{5}}}. \end{array}$$

Thus, the equation

(69) $$q_{2}F\frac{dF}{d\xi }+\frac{q_{5}q_{2}}{q_{4}}\frac{dF}{d\xi }+q_{4}F^{3}+q_{5}F^{2}+\frac{q_{4}q_{7}}{q_{5}}F+q_{7}=0,$$

has the exact solution

(70) $$\begin{array}{c} F=p_{0}+\frac{p_{1}}{a}\sqrt{-\frac{q_{4}q_{7}}{q_{2}q_{5}}}\tanh\left[a\;\sqrt{-\frac{q_{4}q_{7}}{q_{2}q_{5}}}\;\left(\xi +\xi_{0}\right)\right]-\frac{p_{2}q_{4}q_{7}}{a^{2}q_{2}q_{5}}\tanh^{2}\left[a\;\sqrt{-\frac{q_{4}q_{7}}{q_{2}q_{5}}}\;\left(\xi +\xi_{0}\right)\right]. \end{array}$$

All nonlinear partial differential equations that can be reduced to (69) have the solution (70).

We can continue the process by taking, for example, the equation of Bernoulli as the simple equation. The interested reader is directed to [483] for more details on this.

## 6. The SEsM and the Method of Hirota and Inverse Scattering Transform Method

The SEsM can be connected to other famous methods for obtaining exact solutions of nonlinear partial differential equations such as the method of Hirota and the Inverse Scattering Transform (IST) method. Below, we discuss the connection between the SEsM and the method of Hirota and between the IST and the SEsM for the cases of the Korteweg-de Vries equation and the nonlinear Scrödinger equation.

### 6.1. The SEsM and the Method of Hirota

The Hirota method [136,137,138,139,140,141] is a simple and popular method for obtaining multisoliton solutions of nonlinear PDEs, which are integrable. Usually, in Step 1 of this method, a transformation of the solved NPDE is made. Frequently, the form of this transformation is

(71) $$u\left(x,t\right)=2\frac{\partial^{2}}{\partial x^{2}}f\left(x,t\right)=2\left(\frac{f\frac{\partial^{2}f}{\partial x^{2}}-{\left(\frac{\partial f}{\partial x}\right)}^{2}}{f^{2}}\right).$$

In Step 2 of the method, one searches for a solution in the form

(72) $$f=\alpha +\epsilon f_{1}+\epsilon^{2}f_{2}+\epsilon^{3}f_{3}+\ldots .$$

where α and ϵ are parameters. Solution (72) is substituted in the solved nonlinear PDE. In Step 3 of the method, one solves the obtained equations for the orders ϵ, $\epsilon^{2}$, $\epsilon^{3}$, …. In Step 4 of the method, the solution is constructed based on the solutions of the equations for the different powers of ϵ.

Hirota introduced the bilinear operators, which can be written as [497]

(73) $$\begin{array}{ccc} D_{x}^{m}\left(a.b\right) & = & \sum_{j=0}^{m}\frac{{\left(-1\right)}^{\left(m-j\right)}m!}{j!(m-j)!}\frac{\partial^{j}a}{\partial x^{j}}\frac{\partial^{m-j}b}{\partial x^{m-j}},\\ D_{x}^{m}D_{t}^{n}\left(a.b\right) & = & \sum_{j=0}^{m}\sum_{i=0}^{n}\frac{{\left(-1\right)}^{\left(m+n-j-i\right)}m!}{j!(m-j)!}\frac{n!}{i!(n-i)!}\frac{\partial^{i+j}a}{\partial t^{i}\partial x^{j}}\frac{\partial^{m+n-i-j}b}{\partial t^{n-i}\partial x^{m-j}}. \end{array}$$

These operators allow bilinear operators to have useful properties such as finding a solution for the sequence of equations for the powers of ϵ in an effective way.

The Hirota method is connected to the SEsM in the following way [460]:

**Proposition** **3.** *The method of Hirota is connected to a specific case of the SEsM where the transformation in Step 1 of the SEsM is the same as the transformation used in the Hirota method, the representation of function f using $f_{1},f_{2},\ldots$ from Step 2 of the SEsM is (72), the differential equations for $f_{1},f_{2},\ldots$ from Step 3 of the SEsM are the chain of equations obtained for the orders ϵ, $\epsilon^{2}$, … within the scope of the Hirota method, and the simple equations that are used in the construction of the solutions for $f_{1},f_{2},\ldots$ are differential equations for exponential functions.*

One example of the demonstration of the connection between the SEsM and the method of Hirota is in obtaining the three-soliton solution of the Korteweg-de Vries equation:

(74) $$\frac{\partial u}{\partial t}+6u\frac{\partial u}{\partial x}+\frac{\partial^{3}u}{\partial x^{3}}=0.$$

Step 1 of the SEsM is to transform the nonlinearity of this equation using (71). We obtain

(75) $$f\frac{\partial^{2}f}{\partial x\partial t}+\frac{\partial f}{\partial x}\frac{\partial f}{\partial t}+f\frac{\partial^{4}f}{\partial x^{4}}-4\frac{\partial f}{\partial x}\frac{\partial^{3}f}{\partial x^{3}}+3{\left(\frac{\partial^{2}f}{\partial x^{2}}\right)}^{2}=0,$$

which can be written as $\left(D_{x}D_{t}+D_{x}^{4}\right)\left(f\cdot f\right)=0$. Step 2 of the SEsM is to fix the composite function for the solution of the equation. This is made by (72). The substitution of (72) in (75) leads to the differential equations for $f_{1}$, $f_{2}$, …:

(76) $$2\frac{\partial }{\partial x}\left(\frac{\partial }{\partial t}+\frac{\partial^{3}}{\partial x^{3}}\right)f_{1}=0,$$

(77) $$2\frac{\partial }{\partial x}\left(\frac{\partial }{\partial t}+\frac{\partial^{3}}{\partial x^{3}}\right)f_{2}=-D_{x}\left(D_{t}+D_{x}^{3}\right)\left(f_{1}\cdot f_{1}\right),$$

(78) $$2\frac{\partial }{\partial x}\left(\frac{\partial }{\partial t}+\frac{\partial^{3}}{\partial x^{3}}\right)f_{3}=-D_{x}\left(D_{t}+D_{x}^{3}\right)\left(f_{1}\cdot f_{2}+f_{2}\cdot f_{1}\right),$$

etc. The next step of the SEsM is to choose the simple equations for $f_{1}$, $f_{2}$, …. These are equations for exponential functions. One starts with $\frac{dg_{1}}{d\eta_{1}}=g_{1};\;\;g_{1}=\exp\left(\eta_{1}\right),$ where $\eta_{1}=\lambda_{1}x+\omega_{1}t+\sigma_{1}$ and $\lambda_{1}$, $\omega_{1}$ and $\sigma_{1}$ are parameters and chooses $f_{1}$ as

(79) $$f_{1}=\exp\left(\eta_{1}\right).$$

The substitution of (79) in (76) leads to the dispersion relation $\omega_{1}+\lambda_{1}^{3}=0$. $f_{1}$ satisfies (77) and $f_{2}$ can be taken to be 0. $f_{3},f_{4},\ldots$ can also be set to 0. One obtains

(80) $$f=1+\epsilon f_{1}.$$

The parameter ϵ is absorbed by $\sigma_{1}$ and the solution for *f* corresponds to the single soliton solution for *u* by the use of (71).

For obtaining the two-soliton solution of the Korteweg-de Vries equation, one uses the solution of (76) constructed by the solutions of two simple equations $\frac{dg_{1}}{d\eta_{1}}=g_{1};\;\;g_{1}=\exp\left(\eta_{1}\right);\;\;\;\;\frac{dg_{2}}{d\eta_{2}}=g_{2};\;\;g_{2}=\exp\left(\eta_{2}\right).$ Here, $\eta_{i}=\lambda_{i}x+\omega_{i}t+\sigma_{i}$ and $\lambda_{i}$, $\omega_{i}$ and $\sigma_{i}$, i=1,2 are parameters. Then,

(81) $$f_{1}=\exp\left(\eta_{1}\right)+\exp\left(\eta_{2}\right).$$

Two algebraic equations occur from the substitution of (81) in (76): $\omega_{i}+\lambda_{i}^{3}=0,\;\;i=1,2.$ The substitution of (81) in (77) leads to an equation for $f_{2}$, for which the solution is $f_{2}=a_{12}\exp\left(\eta_{1}+\eta_{2}\right),$ with $a_{12}=\frac{{\left(\lambda_{1}-\lambda_{2}\right)}^{2}}{{\left(\lambda_{1}+\lambda_{2}\right)}^{2}}$. $f_{3}$, $f_{4}$, etc., can be set to zero and one arrives at the two-soliton solution of the KdV equation

(82) $$f=1+\epsilon \left[\exp\left(\eta_{1}\right)+\exp\left(\eta_{2}\right)\right]+\epsilon^{2}a_{12}\exp\left(\eta_{1}+\eta_{2}\right).$$

Here, ϵ and $\epsilon^{2}$ are absorbed by $\sigma_{1,2}$ and $a_{1,2}$.

For obtaining the three-soliton solution of the Korteweg-de Vries equation, one needs a solution for $f_{1}$ constructed using the solutions of three simple equations for the exponential functions $\frac{dg_{i}}{d\eta_{i}}=g_{i};\;\;g_{i}=\exp\left(\eta_{i}\right);\;\;i=1,2,3$. Here, $\eta_{i}=\lambda_{i}x+\omega_{i}t+\sigma_{i}$ and $\lambda_{i}$, $\omega_{i}$ and $\sigma_{i}$, i=1,2,3 are parameters. $f_{1}$ is the sum of the solutions of the three simple equations

(83) $$f_{1}=\exp\left(\eta_{1}\right)+\exp\left(\eta_{2}\right)+\exp\left(\eta_{3}\right).$$

The substitution of (83) in (76) leads to three dispersion relations $\omega_{i}+\lambda_{i}^{3}=0,\;\;i=1,2,3.$ For $f_{2}$, after a substitution of (83) in (77), one obtains $f_{2}=a_{12}\exp\left(\eta_{1}+\eta_{2}\right)+a_{13}\exp\left(\eta_{1}+\eta_{3}\right)+a_{23}\exp\left(\eta_{2}+\eta_{3}\right),$ with $a_{ij}=\frac{{\left(\lambda_{i}-\lambda_{j}\right)}^{2}}{{\left(\lambda_{i}+\lambda_{j}\right)}^{2}},\;\;i,j=1,2,3,\;\;i<j.$ The substitution of $f_{1}$ and $f_{2}$ in (78) leads to the following relationship for $f_{3}$: $f_{3}=b_{123}\exp\left(\eta_{1}+\eta_{2}+\eta_{3}\right),\;\;b_{123}=a_{12}a_{13}a_{23}.$ This leads to $f_{4}=0$, $f_{5}=f_{6}=\ldots =0$. The solution for *f* is

(84) $$\begin{array}{c} f=1+\epsilon \left[\exp\left(\eta_{1}\right)+\exp\left(\eta_{2}\right)+\exp\left(\eta_{3}\right)\right]+\epsilon^{2}[a_{12}\exp\left(\eta_{1}+\eta_{2}\right)+\\ a_{13}\exp\left(\eta_{1}+\eta_{3}\right)+a_{23}\exp\left(\eta_{2}+\eta_{3}\right]+\epsilon^{3}b_{123}\exp\left(\eta_{1}+\eta_{2}+\eta_{3}\right), \end{array}$$

and this leads to the three-soliton solution of the Korteweg-de Vries equation.

### 6.2. The SEsM and the Inverse Scattering Transform Method: The Case of the Korteweg-de Vries Equation

Due to the excellent work of Rosales [498,499], we can easily connect the SEsM to various equations that are solvable by the Inverse Scattering Transform method. We demonstrate this for the Korteweg-de Vries equation and the nonlinear Schrödinger equation. We consider the KdV equation

(85) $$\frac{\partial u}{\partial t}+6u\frac{\partial u}{\partial x}+\frac{\partial^{3}u}{\partial x^{3}}=0.$$

We skip Step 1 of the SEsM (the transformation of the nonlinearity). In Step 2 of the SEsM, we present u(x,t) as a composite function of other functions $u_{1},u_{2},\ldots$

(86) $$u\left(x,t\right)=\sum_{n=1}^{\infty }\epsilon^{n}u_{n}\left(x,t\right).$$

Initially, one treats ϵ as a small parameter. The substitution of (86) in (85) leads to equations for $u_{1},u_{2},\ldots$ as follows:

(87) $$\frac{\partial u_{n}}{\partial t}+\frac{\partial^{3}u_{n}}{\partial x^{3}}=-6\frac{\partial }{\partial x}\sum_{j=1}^{n-1}u_{j}u_{n-j},n=2,3,\ldots$$

and the equation for $u_{1}$ is

(88) $$\frac{\partial u_{1}}{\partial t}+\frac{\partial^{3}u_{1}}{\partial x^{3}}=0.$$

The solutions of the obtained equations for $u_{1}$, $u_{2}$, … are connected through a Fourier series to the solution of the simple equation for exponential functions. For $u_{1}$ we use

(89) $$u_{1}=\int_{C}d\lambda \left(k\right)\;\left(-k\right)\exp\left[ikx+k^{3}t\right],$$

where dλ(k) is an appropriate measure in the complex plane C and the term (−k) is introduced for convenience. The form of $u_{1}$ influences the relationships for the equations for $u_{2}$, $u_{3}$, …. One obtains

(90) $$\begin{array}{c} \frac{\partial u_{2}}{\partial t}+\frac{\partial^{3}u_{2}}{\partial x^{3}}=-6i\int_{C^{2}}d\lambda \left(k_{1}\right)d\lambda \left(k_{2}\right)\;\;k_{1}k_{2}\left(k_{1}+k_{2}\right)\exp\left\{i\left[\left(k_{1}+k_{2}\right)x+\left(k_{1}^{3}+k_{2}^{3}\right)t\right]\right\}, \end{array}$$

(91) $$\begin{array}{c} \frac{\partial u_{3}}{\partial t}+\frac{\partial^{3}u_{3}}{\partial x^{3}}=-6\frac{\partial }{\partial x}\left(u_{1}u_{2}+u_{2}u_{3}\right)=\\ =-6i\int_{C^{3}}d\lambda \left(k_{1}\right)d\lambda \left(k_{2}\right)d\lambda \left(k_{3}\right)\;\;\left(k_{1}+k_{2}\right)\left(k_{1}+k_{2}+k_{3}\right)\exp\left\{i\left[\left(k_{1}+k_{2}+k_{3}\right)x+\left(k_{1}^{3}+k_{2}^{3}+k_{3}^{3}\right)t\right]\right\}. \end{array}$$

The solutions for $u_{2},u_{3},\ldots$ are assumed to be of a form similar to that of $u_{1}$:

(92) $$u_{n}\left(x,t\right)=\int_{C^{n}}{\left[d\lambda \left(k\right)\right]}^{n}\Phi_{n}\left(k_{1},\ldots ,k_{n}\right)\exp\left\{i\Omega_{n}\right\}.$$

The substitution of (92) in the equations for $u_{2},u_{3},\ldots$ leads to the fixation of $\Phi_{n}$ and $\Omega_{n}$. The fixation leads to

(93) $$\begin{array}{c} \Phi_{2}\left(k_{1},k_{2}\right)=1;\;\;\Omega_{2}=\left(k_{1}+k_{2}\right)x+\left(k_{1}^{3}+k_{2}^{3}\right)t,\\ \Phi_{3}\left(K_{1},k_{2},k_{3}\right)=-\frac{k_{1}+k_{2}+k_{3}}{\left(k_{1}+k_{2}\right)\left(k_{2}+k_{3}\right)};\;\;\Omega_{3}=\left(k_{1}+k_{2}+k_{3}\right)x+\left(k_{1}^{2}+k_{2}^{2}+k_{3}^{2}\right)t,\\ \Phi_{4}=\frac{k_{1}+k_{2}+k_{3}+k_{4}}{\left(k_{1}+k_{2}\right)\left(k_{2}+k_{3}\right)\left(k_{3}+k_{4}\right)};\;\;\Omega_{4}=\left(k_{1}+k_{2}+k_{3}+k_{4}\right)x+\left(k_{1}^{2}+k_{2}^{2}+k_{3}^{2}+k_{4}^{2}\right)t. \end{array}$$

This leads to the following relationship for $u_{n}$

(94) $$u_{n}={\left(-1\right)}^{n+1}i\frac{\partial }{\partial x}\int_{C^{n}}{\left[d\lambda \left(k\right)\right]}^{n}\frac{\exp(i\Omega_{n})}{\prod_{j=1}^{n-1}\left(k_{j}+k_{j+1}\right)},$$

and the solution of the Korteweg-de Vries equation is

(95) $$u=i\frac{\partial }{\partial x}\left[\sum_{n=1}^{\infty }{\left(-\epsilon \right)}^{n}\int_{C^{n}}{\left[d\lambda \left(k\right)\right]}^{n}\frac{\exp(i\Omega_{n})}{\prod_{j=1}^{n-1}\left(k_{j}+k_{j+1}\right)}\right].$$

One rewrites (95) as

(96) $$u\left(x,t\right)=\frac{\partial }{\partial x}\sum_{n=1}^{n}{\left(-\epsilon \right)}^{n}\int_{C^{n}}d{\left[\lambda \left(k\right)\right]}^{n}\;\;\hat{p}\left(k_{1}\right)\hat{P}\left(k_{1},k_{2}\right)\hat{P}\left(k_{2},k_{3}\right),\ldots \hat{P}\left(k_{n-1},k_{n}\right)\hat{p}\left(k_{n}\right),$$

where ${\hat{p}}_{k}=\exp\left\{\frac{i}{2}\left[kx-\omega \left(k\right)t\right]\right\};\;\;\hat{P}\left(k,q\right)=i\frac{\hat{p}\left(k\right)\hat{p}\left(q\right)}{k+q};\;\;\omega \left(k\right)=-k^{3}$. Two possibilities exist for the measure dλ(k), discrete or continuous. For the case of discrete dλ(k), the integral can be replaced by a sum: $\int_{C}d\lambda \left(k\right)f\left(k\right)=\sum_{m}a_{m}^{2}f\left(ik_{m}\right)$. Thus,

(97) $$u=\frac{\partial }{\partial x}\sum_{n=1}^{\infty }{\left(-\epsilon \right)}^{n}\sum_{m_{j}}p_{m_{1}}P_{m_{1},m_{2}}P_{m_{2},m_{3}}\ldots P_{m_{n-1},m_{n}}p_{m_{n}}.$$

Here, $P_{mq}=\frac{p_{m}p_{q}}{k_{m}+k_{q}};\;\;p_{m}=a_{m}\hat{p}\left(ik_{m}\right)=a_{m}\exp\left\{\frac{1}{2}\left(-k_{m}x+k_{m}^{3}t\right)\right\}$, and (97) can be written as

(98) $$u=-\epsilon \frac{\partial }{\partial x}\left[p^{T}{\left(I+\epsilon P\right)}^{-1}p\right],$$

where *p* is the column vector of all $p_{m}$, *P* is the square matrix of all $P_{mq}$, and $p^{T}$ is the transpose of *p*. The matrix *P* is real symmetric and positive definite for $k_{m}>0$ and real for $a_{m}$. Thus, *u* is nonsingular for ϵ>0 and ϵ can be absorbed into the coefficients $a_{m}$. Because of this, Solution (98) is not limited to only small values of ϵ.

Solution (98) can be rewritten in a more common form. Observing that $\frac{\partial P}{\partial x}=-\frac{1}{2}pp^{T}$, we arrive at

(99) $$u=2\frac{\partial }{\partial x}\mathrm{Tr}\left[{\left(I+P\right)}^{-1}\frac{\partial P}{\partial x}\right]=2\frac{\partial^{2}}{\partial x^{2}}\mathrm{Tr}\left[\ln\left(I+P\right)\right]=2\frac{\partial^{2}}{\partial x^{2}}\ln\det\left(I+P\right).$$

This is the relationship for the multisoliton solution of the Korteweg-de Vries equation.

For the case of continuous measure dλ(k), we obtain the Gelfand–Levitan–Marchenko equation from the methodology of the Inverse Scattering Transform method as follows. *P* is considered more a general operator than a square matrix: $\left(Pf\right)\left(k\right)=\int_{C}d\lambda \left(l\right)\hat{P}\left(k,l\right)f\left(l\right).$ Thus, the solution of the KdV equation is

(100) $$u=-\epsilon \frac{\partial }{\partial x}\int_{C}d\lambda \left(k\right)\left[\hat{p}{\left(k\right)[\left(I+\epsilon P\right)}^{-1}p\right]\left(k\right).$$

Solution (100) can be written as

(101) $$u=2\frac{\partial }{\partial x}K\left(x,x\right).$$

Here, $K\left(x,y\right)=-\frac{\epsilon }{2}p^{T}\left(x\right){\left[I+\epsilon P\left(x\right)\right]}^{-1}p\left(y\right)$, and after a calculation, one obtains

(102) $$K\left(x,y\right)=-\frac{\epsilon }{2}p^{T}\left(x\right)p\left(y\right)-\frac{\epsilon }{2}\int_{x}^{\infty }dzK\left(x,z\right)p^{T}\left(z\right)p\left(y\right).$$

This is the GLM equation with

(103) $$B\left(x+y\right)=\frac{\epsilon }{2}p^{T}\left(x\right)p\left(y\right)=\frac{\epsilon }{2}\int d\lambda \left(k\right)\exp\left[i\left(\frac{k}{2}\left(x+y\right)+k^{3}t\right)\right].$$

### 6.3. The SEsM and the Inverse Scattering Transform Method: The Case of the Nonlinear Schrödinger Equation

We consider the nonlinear Schrödinger equation

(104) $$i\frac{\partial \varphi }{\partial t}+\frac{\partial^{2}\varphi }{\partial x^{2}}+2\sigma |\varphi {|}^{2}\varphi =0.$$

Step 1 of the SEsM (the transformation of the nonlinearity) is skipped. In Step 2 of the SEsM, we present φ(x,t) as a composite function similar to (86). Performing the calculations for the solutions of the equations for the components of φ using differential equations for exponential functions as simple equations (Steps 3 and 4 of the SEsM) we arrive at

(105) $$\varphi =\sum_{n=0;m=2n+1}d\lambda_{1}d\mu_{2}d\lambda_{3}d\mu_{4}\ldots d\lambda_{m}\sigma^{n}\epsilon^{m}\int_{C^{m}}\frac{\exp(i\Omega_{m})}{\prod_{j=1}^{2n}\left(k_{j}+k_{j+1}\right)}.$$

In (105), $\Omega_{n}\sum_{1}^{n}\left[k_{j}x+{\left(-1\right)}^{j}k_{j}^{2}t\right]$. dλ(k) and dμ(k) are measures such that $d\nu^{*}\left(-k^{*}\right)=d\lambda \left(k\right)$ (* denotes complex conjugation). $d\lambda_{j}=d\lambda \left(k_{j}\right)$; $d\mu_{j}=d\mu \left(k_{j}\right)$. There is a quantity analogous to *P* from the case of the Korteweg-de Vries equation above. The definition of this quantity for the case of the nonlinear Schrödinger equation is

(106) $$\hat{P}\left(k,l\right)=i\frac{{\hat{p}}^{*}\left(k\right)\hat{p}\left(l\right)}{-k^{*}+l}.$$

In $\Omega_{n}$, one has two dispersion relationships: $\omega_{1}\left(k\right)=k^{2}$ and $\omega_{2}\left(k\right)=-k^{2}$. In $\hat{p}$, we take the first of them. Denoting the conjugate operator of *P* as $\bar{P}$, one can rewrite (105) as

(107) $$\varphi =\epsilon \int_{C}d\lambda \left(k\right)\;\;\hat{p}\left(k\right)\left\{{\left(I+\sigma \epsilon^{2}\bar{P}P\right)}^{-1}\right\}\left(k\right)=\epsilon p^{T}{\left(I+\sigma \epsilon^{2}\bar{P}P\right)}^{-1}p.$$

For $|\varphi {|}^{2}$, one obtains

(108) $${|\varphi |}^{2}=-\epsilon^{2}\frac{\partial }{\partial x}p^{T}{\left(I+\sigma \epsilon^{2}\bar{P}P\right)}^{-1}\bar{P}\bar{p}.$$

This is reduced to the relationship of Zakharov and Shabat [500] for the multiple-envelope solutions of the nonlinear Schrödinger equation for the specific case of (108) of discrete measure (discrete λ(k)) and when *p* is a vector and *P* is a square matrix. In this case,

(109) $${|\varphi |}^{2}=\frac{1}{\sigma }\frac{\partial^{2}}{\partial x^{2}}\ln\det\left(I+\sigma \epsilon^{2}\bar{P}P\right).$$

## 7. Special Application: The SEsM and the SIR Model of Epidemics

Below we discuss the exact solutions of an equation. This equation is obtained based on the SIR model in epidemiology. The obtained solutions can describe epidemic waves caused by different diseases (COVID-19-inclusive).

The equation is obtained using the classic idea of Kermak and McKendrick [501] for the transformation of the SIR model with constant coefficients to a single nonlinear differential equation. We consider an epidemic in a population. The population is divided into three groups: susceptible individuals—*S*; infected individuals—*I*; recovered individuals—*R*. The model equations for the time changes in the numbers of individuals in the above three groups are

(110) $$\begin{array}{ccc} \frac{dS}{dt} & = & -\frac{\tau }{N}SI,\\ \frac{dI}{dt} & = & \frac{\tau }{N}SI-\rho I,\\ \frac{dR}{dt} & = & \rho I. \end{array}$$

In (refsir1), τ is the transmission rate and ρ is the recovery rate. These rates are assumed to be constants.

(111) N=S+I+R,

is the total population, which is assumed to be constant. The model (110) can be reduced to a single equation for *R* as follows. From the last equation of (110) we have

(112) $$I=\frac{1}{\rho }\frac{dR}{dt}.$$

The substitution of (112) in the first equation of (110) leads to the relationship

(113) $$S=S\left(0\right)\exp\left\{-\frac{\tau }{\rho N}\left[R-R\left(0\right)\right]\right\}.$$

Here, S(0) and R(0) are the numbers of susceptible and recovered individuals at the time t=0. The substitution of (111) and (113) in the last equation of (110) leads to the differential equation for *R*

(114) $$\frac{dR}{dt}=\rho \left\{N-R-S\left(0\right)\exp\left[-\frac{\tau }{\rho N}\left(R-R\left(0\right)\right)\right]\right\}.$$

Below, we assume R(0)=0 (no recovered individuals at t=0). Let us consider the ratio $\frac{\tau R}{\rho N}$. The maximum of the ratio R/N is 1 and if τ<<ρ, then $\frac{\tau R}{\rho N}<<1$. Let us consider a kind of epidemic, where τ<<ρ (transmission rate is much lower than the recovery rate). Then, $\exp\left[-\frac{\tau }{\rho N}R\right]$ can be represented as a Taylor series

(115) $$\exp\left[-\frac{\tau }{\rho N}R\right]=\sum_{j=0}^{M}{\left(-\frac{\tau }{\rho N}R\right)}^{j}.$$

*M* has infinite value in the full Taylor series but we can truncate it at M=2, M=3,..., if $-\frac{\tau }{\rho N}R$ is small enough. From (114) we obtain

(116) $$\frac{dR}{dt}=\rho \left\{N-R-S\left(0\right)\sum_{j=0}^{M}{\left(-\frac{\tau }{\rho N}R\right)}^{j}\right\},\;\;\;M=2,3,\ldots$$

We apply the most simple version of the SEsM to (116). The solution is searched as a composite function of a single function *U* and the form of the composite function is a polynomial

(117) $$R=\sum_{k=0}^{K}\mu_{k}U^{k},$$

where $\mu_{l}$ and *K* are parameters. We also fix the form of the simple equation for *U*

(118) $$\frac{dU}{dt}=\sum_{l=0}^{L}\nu_{l}U^{l},$$

where $\nu_{l}$ and *L* are parameters.

In order to proceed with the application of the SEsM, we have to obtain a balance equation that connects the parameters *M*, *K*, and *L*. The form of this balance equation is

(119) L−1=K(M−1).

Then, we proceed as follows. We fix the value of *M*. This fixes the form of (116). Then, we fix the value of *K*. This fixes the form of the composite function for the solution of the equation. Then, from (119) we obtain the value of *L*. The fixation of the parameters *M*, *K*, and *L* and the substitution of (117) and (118) in (116) lead us to the system of nonlinear algebraic equations as required by the algorithm of the SEsM. The nontrivial solutions of this system lead to solutions of *R*. The substitution of these solutions in (112) leads to the corresponding solutions for *I*. Finally, the solutions for *R* and *I* are substituted in (111) and we obtain the solution for *S*.

Below, we consider the simplest case M=2. This is the classical Kermak–McKendrick case. From (116), the equation we have to solve is

(120) $$\frac{dR}{dt}=\alpha R^{2}+\beta R+\gamma ,$$

where γ=ρ[N−S(0)]; $\beta =\frac{\tau S(0)}{N}-\rho$; $\alpha =-\frac{\tau^{2}S\left(0\right)}{\rho N^{2}}$, and (120) is an equation of the Riccati kind. From (119),

(121) L=K+1.

Let us consider the case K=1, L=2. In this case,

(122) $$R=\mu_{0}+\mu_{1}U,$$

and the simple equation is

(123) $$\frac{dU}{dt}=\nu_{0}+\nu_{1}U+\nu_{2}U^{2}.$$

The substitution of (122) and (123) in (120) leads to a system of nonlinear algebraic equations. One nontrivial solution of this system is $\mu_{0}=0$, $\mu_{1}=1$; $\nu_{0}=\gamma$; $\nu_{1}=\beta$, $\nu_{2}=\alpha$. The simple equation, Equation (123), coincides with (120). As we consider the case τ<<ρ then, $\beta^{2}-4\alpha \gamma \approx \rho \left[\rho -\frac{2\tau S(0)}{N}\right]>0$. This is because S(0)<<N. In addition, we have $\beta^{2}-4\alpha \gamma =\frac{\tau^{2}S{\left(0\right)}^{2}}{N^{2}}\left(1+4\frac{N-S(0)}{S(0)}\right)>\frac{\tau^{2}S{\left(0\right)}^{2}}{N^{2}}{\left(1-\frac{2\tau R}{\rho N}\right)}^{2}={\left(2\alpha R+\beta \right)}^{2}$. A particular solution of (120) is

(124) $$R\left(t\right)=-\frac{\beta }{2\alpha }-\frac{\theta }{2\alpha }\tanh\left[\frac{\theta (t+C)}{2}\right],$$

where $\theta^{2}=\beta^{2}-4\alpha \gamma >0$ and *C* is a constant of integration. The substitution of (124) in (112) leads to the well-known bell-shaped curve for the evolution of the number of infected individuals *I*

(125) $$I=-\frac{\theta^{2}}{2\alpha \rho }{\mathrm{sech}}^{2}\left[\frac{\theta (t+C)}{2}\right].$$

Note that $-\frac{\theta^{2}}{2\alpha \rho }$ is positive as α is negative.

We know a particular solution (124) of (120). Then, we can write the general solution of (120) as $R=-\frac{\beta }{2\alpha }-\frac{\theta }{2\alpha }\tanh\left[\frac{\theta (t+C)}{2}\right]+\frac{D}{v}$, where *D* is a constant and v(t) is the solution of the linear differential equation

(126) $$\frac{dv}{dt}-\theta \tanh\left[\frac{\theta (t+C)}{2}\right]v=-\alpha D.$$

The solution of (126) is

(127) $$v=\cosh^{2}\left[\frac{\theta (t+C}{2}\right]\left\{E-\frac{2\alpha D}{\theta }\tanh\left[\frac{\theta (t+C}{2}\right]\right\},$$

where *E* is a constant of integration. Then, the general solution of Equation (120) is

(128) $$R\left(t\right)=-\frac{\beta }{2\alpha }-\frac{\theta }{2\alpha }\tanh\left[\frac{\theta (t+C)}{2}\right]+\frac{D}{\cosh^{2}\left[\frac{\theta (t+C}{2}\right]\left\{E-\frac{2\alpha D}{\theta }\tanh\left[\frac{\theta (t+C}{2}\right]\right\}}.$$

This solution introduces corrections to the well-known curve for the time evolution of recovered persons within the scope of the SIR model. The substitution of (128) in (112) leads to the following relationship for the time evolution of the number of infected persons:

(129) $$I=-\frac{\theta^{2}}{2\alpha \rho }{\mathrm{sech}}^{2}\left[\frac{\theta (t+C)}{2}\right]-\frac{D}{\rho }\left\{-\frac{\theta E\sinh\left[\theta (t+C)\right]-\alpha D\cosh\left[\theta \left(t+C\right)\right]}{\cosh^{4}\left[\frac{\theta (t+C)}{2}\right]{\left\{E-\frac{2\alpha D}{\theta }\tanh\left[\frac{\theta (t+C)}{2}\right]\right\}}^{2}}\right\}.$$

This solution leads to corrections to the well-known bell-shaped curve for the time evolution of infected individuals in the SIR model. For example, let t=−C. Then, from (125),

(130) $$I=-\frac{\theta^{2}}{2\alpha \rho },$$

and from (129),

(131) $$I=-\frac{\theta^{2}}{2\alpha \rho }-\frac{\alpha D^{2}}{\rho E}.$$

The presence of the general solution (128) of (116) makes the further consideration of the subcases of the case M=2 unnecessary. What we can obtain by considering these cases are the specific cases of the general solution (128).

## 8. Other Examples of the Application of the SEsM

The SEsM and its most simple version, the MMSE, have numerous applications. Below, we mention several results. In most cases, we search for traveling wave solutions of the kind u(x,t)=u(ξ)=u(x−vt), and the composite function from Step 2 of the SEsM is given by the power series of the solution *V* of the simple equation

(132) $$u\left(\xi \right)=\sum_{\mu =-\nu }^{\eta }\theta_{\mu }{\left[V\left(\xi \right)\right]}^{\mu }.$$

In (132), ν>0, μ>0, $\theta_{\mu }$ are parameters. In most cases, we use as the simple equation

(133) $$\frac{dV}{d\xi }=\sum_{\alpha =0}^{\beta }\gamma_{\alpha }V{\left(\xi \right)}^{\alpha },$$

where $\gamma_{\alpha }$ is a parameter. Equation (133) has two important specific cases: the equations of Bernoulli and Riccati. Their solutions are expressed by elementary functions. For the Bernoulli equation $\gamma_{0}=0$; $\gamma_{1}=a$; $\gamma_{2}=\cdots =\gamma_{k-1}=0$; $\gamma_{k}=b$; $\gamma_{k+1}=\cdots =\gamma_{\beta }=0$. The equation is

(134) $$\frac{dV}{d\xi }=aV\left(\xi \right)+b{\left[V\left(\xi \right)\right]}^{k},$$

where *k* is an integer and k>1. The solutions that are used are

(135) $$\begin{array}{c} V\left(\xi \right)=\sqrt[{k-1}]{\frac{a\exp[a\left(k-1\right)\left(\xi +\xi_{0}\right)]}{1-b\exp[a\left(k-1\right)\left(\xi +\xi_{0}\right)]}},\;\;b<0,\;\;a>0;\\ V\left(\xi \right)=\sqrt[{k-1}]{-\frac{a\exp[a\left(k-1\right)\left(\xi +\xi_{0}\right)]}{1+b\exp[a\left(k-1\right)\left(\xi +\xi_{0}\right)]}},\;\;b>0,\;\;a<0. \end{array}$$

$\xi_{0}$ is a constant of integration.

For the Riccati equation, $\gamma_{0}=c$; $\gamma_{1}=b$; $\gamma_{2}=a$, $\gamma_{3}=\cdots =\gamma_{\beta }=0$. The equation is

(136) $$\frac{dV}{d\xi }=a{\left[V\left(\xi \right)\right]}^{2}+bV\left(\xi \right)+c.$$

The solution that is used is

(137) $$V\left(\xi \right)=-\frac{b}{2a}-\frac{\theta }{2a}\tanh\left[\frac{\theta (\xi +\xi_{0})}{2}\right].$$

Here, $\theta^{2}=b^{2}-4ac>0$ and $\xi_{0}$ is a constant of integration.

As a specific case of the use of the equation of Riccati, we also consider the extended tanh-function equation

(138) $$\frac{dV}{d\xi }={\bar{c}}^{2}-{\bar{a}}^{2}V^{2}.$$

Equation (138) is obtained from (136) when b=0, $a=-{\bar{a}}^{2}$, and $c={\bar{c}}^{2}$. The solution of (138) that is used is

(139) $$V\left(\xi \right)=\frac{\bar{c}}{\bar{a}}\tanh\left[\bar{a}\;\bar{c}\left(\xi +\xi_{0}\right)\right],$$

where ${\bar{a}}^{2}V{\left(\xi \right)}^{2}<{\bar{c}}^{2}$ and $\xi_{0}$ is a constant of integration.

The first illustration of the methodology of the SEsM is for obtaining traveling wave solutions of the class of equations

(140) $$\sum_{p=1}^{N_{1}}\alpha_{p}\frac{\partial^{p}Q}{\partial t^{p}}+\sum_{q=1}^{N_{2}}\beta_{q}\frac{\partial^{q}Q}{\partial x^{q}}+\sum_{m=1}^{M}\mu_{m}Q^{m}=0.$$

In (140), $\alpha_{p}$, $\beta_{q}$, and $\mu_{m}$ are parameters. Specific cases of (140) are used as model equations in the theory of the migration of populations. If $N_{1}=1$, $N_{2}=2$, $\alpha_{1}=1$, $\beta_{1}=0$, $\beta_{2}=-1$, (140) is reduced to an equation of the kind $\frac{\partial Q}{\partial t}-\frac{\partial^{2}Q}{\partial x^{2}}=f\left(Q\right),$ with $f\left(Q\right)=-\sum_{m=1}^{M}\mu_{m}Q^{m}$. Such kinds of equations are widely used in the modeling of genetic diffusion.

One searches for traveling wave solutions Q(x,t)=Q(ξ)=Q(x−vt). Taking into account that $N=\max(N_{1},N_{2})$, one can extend (140) to

(141) $$\sum_{n=1}^{N}\left(\alpha_{n}\frac{\partial^{n}Q}{\partial t^{n}}+\beta_{n}\frac{\partial^{n}Q}{\partial x^{n}}\right)+\sum_{m=1}^{M}\mu_{m}Q^{m}=0,$$

where the coefficients of the additional terms are equal to 0. The traveling wave solution and $\nu_{n}=\beta_{n}+\alpha_{n}{\left(-v\right)}^{n}$ reduce (141) to

(142) $$\sum_{n=1}^{N}\nu_{n}\frac{d^{n}Q}{d\xi^{n}}+\sum_{m=1}^{M}\mu_{m}Q^{m}=0.$$

As the simple equation, we use the equation of Bernoulli (134). The substitution of (132) and (134) in (142) leads to a polynomial $P=\kappa_{r}\phi^{r}+\kappa_{r-1}\phi^{r-1}+\cdots +\kappa_{0}=0$. In *P*, *r* is an integer. The coefficients κ depend on the parameters of the solved equation and the parameters of the solution. The solution is obtained when all coefficients are set to 0. The result is a system of nonlinear algebraic relationships

(143) $$\kappa_{l}=0,\;l=r,r-1,\ldots ,0$$

Next, we move to Step 3 of the SEsM, ensuring that each equation from (143) has at least two terms by balancing the highest powers arising in (142). This leads to the following balance equation (for the case when the Bernoulli equation is used as the simple equation): LM=L+N(k−1). Thus, the choice of the highest power *L* of the solution polynomial leads to the fixation of the parameter *k* for the Bernoulli equation

(144) $$k=1+\frac{L(M-1)}{N}.$$

If the Riccati equation is used as the simple equation, then the balance equation is L(M−1)=N.

Several examples of exact solutions follow for cases where the Bernoulli equation is used as the simple equation. We first set N=2, M=2, and (142) becomes

(145) $$\nu_{1}\frac{d^{2}Q}{d\xi^{2}}+\nu_{2}\frac{dQ}{d\xi }+\mu_{2}Q^{2}+\mu_{1}Q=0.$$

A specific case of this equation is the equation studied by Ablowitz and Zeppetela [502] $\frac{d^{2}Q}{d\xi^{2}}+v\frac{dQ}{d\xi }+Q\left(1-Q\right)=0.$ Ablowitz and Zeppetela obtained the solution $Q\left(\xi \right)=\frac{1}{{\left[1-r\exp\left(\xi /\sqrt{6}\right)\right]}^{2}},\;r<0,\;v=5/\sqrt{6}.$ Another solution of the last equation is obtained using the tanh-method. This solution is [503] $Q\left(\xi \right)=Q\left(x-vt\right)=\frac{1}{4}\left\{1-{\left(\tanh\frac{1}{2\sqrt{6}}\xi \right)}^{2}\right\},v=\frac{5}{\sqrt{6}}.$

We search for a solution of (145) of the kind $Q\left(\xi \right)=\sum_{i=0}^{L}a_{i}\phi^{i};\;\frac{d\phi }{d\xi }=b\phi^{\left(1+L/2\right)}+a\phi$. L=2 is assumed and for this case, in Step 4 of the SEsM one obtains the system of nonlinear algebraic relationships between the parameters of the solutions and the parameters of (142)

(146) $$\begin{array}{ccc} 6\nu_{1}a_{2}b^{2}+\mu_{2}a_{2}^{2} & = & 0,\\ 2\nu_{2}a_{2}b+10\nu_{1}a_{2}ba+2\mu_{2}a_{2}a_{1}+2\nu_{1}a_{1}b^{2} & = & 0,\\ \mu_{1}a_{2}+\mu_{2}a_{1}^{2}+2\nu_{2}a_{2}a+\nu_{2}a_{1}b+4\nu_{1}a_{2}a^{2}+ & & \\ 2\mu_{2}a_{2}a_{0}+3\nu_{1}a_{1}ab & = & 0,\\ \mu_{1}a_{1}+2\mu_{2}a_{1}a_{0}+\nu_{1}a_{1}a^{2}+\nu_{2}a_{1}a & = & 0,\\ \mu_{1}a_{0}+\mu_{2}a_{0}^{2} & = & 0. \end{array}$$

A solution of (146) is $a_{0}=\frac{6}{25}\frac{\nu_{2}^{2}}{\nu_{1}\mu_{2}},\;a_{1}=0,\;a_{2}=-6\frac{\nu_{1}b^{2}}{\mu_{2}},\;\mu_{1}=-\frac{6}{25}\frac{\nu_{2}^{2}}{\nu_{1}}.$ The corresponding solution of (145) is

(147) $$\begin{array}{c} Q\left(\xi \right)=\frac{6}{25}\frac{\nu_{2}^{2}}{\nu_{1}\mu_{2}}-6\frac{\nu_{1}b^{2}}{\mu_{2}}{\left\{\frac{a\exp[a\left(\xi +\xi_{0}\right)]}{1-b\exp[a\left(\xi +\xi_{0}\right)]}\right\}}^{2}. \end{array}$$

for the case when b<0 and a>0. Another solution of (146) is $a_{0}=\frac{a_{1}^{2}}{4a_{2}},\;\nu_{2}=\frac{5\mu_{1}}{6a},\;\mu_{2}=-\frac{4\mu_{1}a_{2}}{a_{1}^{2}},\;b=\frac{2aa_{2}}{a_{1}}.$ The corresponding solution of (142) is

(148) $$\begin{array}{c} Q\left(\xi \right)=a_{2}{\left[\frac{a\exp(a\left(\xi +\xi_{0}\right))}{1-\frac{2aa_{2}}{a_{1}}\exp\left(\xi +\xi_{0}\right)}\right]}^{2}+a_{1}\left[\frac{a\exp(a\left(\xi +\xi_{0}\right))}{1-\frac{2aa_{2}}{a_{1}}\exp\left(\xi +\xi_{0}\right)}\right]+\frac{a_{1}^{2}}{4a_{2}}. \end{array}$$

Next, we set L=4 ( L=3 is impossible as *k* has to be an integer). In Step 3 of the SEsM, we obtain the system of nonlinear algebraic relationships between the parameters

(149) $$\begin{array}{ccc} 24\nu_{1}a_{4}b^{2}+\mu_{2}a_{4}^{2} & = & 0,\\ 2\mu_{2}a_{4}a_{3}+15\nu_{1}a_{3}b^{2} & = & 0,\\ 4\nu_{2}a_{4}b+40\nu_{1}a_{4}ba+8\nu_{1}a_{2}b^{2}+2\mu_{2}a_{4}a_{2}+\mu_{2}a_{3}^{2} & = & 0,\\ 24\nu_{1}a_{3}ba+2\mu_{2}a_{4}a_{1}+3\nu_{1}a_{1}b^{2}+3\nu_{2}a_{3}b+2\mu_{2}a_{3}a_{2} & = & 0,\\ 2\mu_{2}a_{4}a_{0}+16\nu_{1}a_{4}a^{2}+4\nu_{2}a_{4}a+\mu_{1}a_{4}+2\mu_{2}a_{3}a_{1}+\mu_{2}a_{2}^{2}+ & & \\ 2\nu_{2}a_{2}b+12\nu_{1}a_{2}ba & = & 0,\\ \mu_{1}a_{3}+9\nu_{1}a_{3}a^{2}+3\nu_{2}a_{3}a+\nu_{2}a_{1}b+2\mu_{2}a_{2}a_{1}+2\mu_{2}a_{3}a_{0}+ & & \\ 4\nu_{1}a_{1}ba & = & 0,\\ 4\nu_{1}a_{2}a^{2}+\mu_{1}a_{2}+2\mu_{2}a_{2}a_{0}+2\nu_{2}a_{2}a+\mu_{2}a_{1}^{2} & = & 0,\\ \nu_{2}a_{1}a+\nu_{1}a_{1}a^{2}+2\mu_{2}a_{1}a_{0}+\mu_{1}a_{1} & = & 0,\\ \mu_{1}a_{0}+\mu_{2}a_{0}^{2} & = & 0. \end{array}$$

One solution of this system is $a_{0}=\frac{6}{25}\frac{\nu_{2}^{2}}{\nu_{1}\mu_{2}},\;a_{1}=a_{2}=a_{3}=0,\;a_{4}=-24\frac{\nu_{1}b^{2}}{\mu_{2}},\;a=-\frac{1}{10}\frac{\nu_{2}}{\nu_{1}},\mu_{1}=-\frac{6}{25}\frac{\nu_{2}^{2}}{\nu_{1}}.$ The corresponding solution of (145) (b>0, a<0) is

(150) $$Q\left(\xi \right)=\frac{6}{25}\frac{\nu_{2}^{2}}{\nu_{1}\mu_{2}}\left(1-b{\left\{\frac{\exp[-\frac{\nu_{2}\left(\xi +\xi_{0}\right)}{5\nu_{1}}]}{1+b\exp[-\frac{\nu_{2}\left(\xi +\xi_{0}\right)}{5\nu_{1}}]}\right\}}^{2}\right).$$

Another solution of (149) is $a_{4}=\frac{ba_{2}}{2a},\;a_{3}=0,\;a_{1}=0,a_{0}=\frac{aa_{2}}{2b},\;\mu_{1}=\frac{12}{5}a\nu_{2},\;\mu_{2}=-\frac{24}{5}\frac{b\nu_{2}}{a_{2}},\;\nu_{1}=\frac{\nu_{2}}{10a}.$ The corresponding solution of (145) is

(151) $$\begin{array}{c} Q\left(\xi \right)=\frac{ba_{2}}{2a}{\left(\frac{a\exp[2a\left(\xi +\xi_{0}\right)]}{1+b\exp[2a\left(\xi +\xi_{0}\right)]}\right)}^{2}+a_{2}\left(\frac{a\exp[2a\left(\xi +\xi_{0}\right)]}{1+b\exp[2a\left(\xi +\xi_{0}\right)]}\right)+\frac{aa_{2}}{2b}. \end{array}$$

Here, b>0, a<0.

Next, one can set N=2, M=3. The equation that is to be solved is

(152) $$\nu_{1}\frac{d^{2}Q}{d\xi^{2}}+\nu_{2}\frac{dQ}{d\xi }+\mu_{3}Q^{3}+\mu_{2}Q^{2}+\mu_{1}Q=0.$$

This equation is connected to the Huxley equation for gene propagation [504]. Other equations having polynomial nonlinearity of the third order are the Burgers–Huxley equation and equations that model aspects of neuronal activity [505].

Several solutions of (152) are below. For the case L=1, the SEsM leads to a solution of the kind

(153) $$Q\left(\xi \right)=\sum_{i=0}^{L}a_{i}\phi {\left(\xi \right)}^{i},\;\frac{dQ}{d\xi }=b\phi^{1+L}+a\phi .$$

For the case b>0, a<0, the solution is

(154) $$Q\left(\xi \right)=-\frac{b\sqrt{-2\mu_{3}\mu_{1}}}{\mu_{3}}\frac{\left(\frac{2\mu_{2}\nu_{1}-\nu_{2}\sqrt{-2\mu_{3}\nu_{1}}}{3\nu_{1}\sqrt{-2\mu_{3}\nu_{1}}}\right)\exp\left[\left(\frac{2\mu_{2}\nu_{1}-\nu_{2}\sqrt{-2\mu_{3}\nu_{1}}}{3\nu_{1}\sqrt{-2\mu_{3}\nu_{1}}}\right)\left(\xi +\xi_{0}\right)\right]}{1+b\exp[\left(\frac{2\mu_{2}\nu_{1}-\nu_{2}\sqrt{-2\mu_{3}\nu_{1}}}{3\nu_{1}\sqrt{-2\mu_{3}\nu_{1}}}\right)\left(\xi +\xi_{0}\right)]}.$$

For L=2, the solution of (152) for the case a>0, b<0 is

(155) $$Q\left(\xi \right)=\frac{2b\sqrt{-2\nu_{1}\mu_{3}}}{\mu_{3}}\left(\frac{\frac{-\sqrt{2}\nu_{1}\sqrt{\mu_{1}\mu_{3}}-\nu_{2}\sqrt{-\nu_{1}\mu_{3}}}{6\nu_{1}\sqrt{-\nu_{1}\mu_{3}}}\exp\left[2\frac{-\sqrt{2}\nu_{1}\sqrt{\mu_{1}\mu_{3}}-\nu_{2}\sqrt{-\nu_{1}\mu_{3}}}{6\nu_{1}\sqrt{-\nu_{1}\mu_{3}}}\left(\xi +\xi_{0}\right)\right]}{1-b\exp[2\frac{-\sqrt{2}\nu_{1}\sqrt{\mu_{1}\mu_{3}}-\nu_{2}\sqrt{-\nu_{1}\mu_{3}}}{6\nu_{1}\sqrt{-\nu_{1}\mu_{3}}}\left(\xi +\xi_{0}\right)]}\right)-\frac{\sqrt{\mu_{1}\mu_{3}}}{\mu_{3}}.$$

One can continue by assuming N=2,M=4. The equation that has to be solved is

(156) $$\nu_{1}\frac{d^{2}Q}{d\xi^{2}}+\nu_{2}\frac{dQ}{d\xi }+\mu_{4}Q^{4}+\mu_{3}Q^{3}+\mu_{2}Q^{2}+\mu_{1}Q=0.$$

Equations of this kind arise in the theory of the migration of populations [476,477]. The application of the SEsM leads to the conclusion that this equation has solutions of the form $Q\left(\xi \right)=\sum_{i=0}^{L}a_{i}{\left[\phi \left(\xi \right)\right]}^{i},\;\frac{d\phi }{d\xi }=b\phi^{\left(1+3L/2\right)}+a\phi$. For L=2, one possible solution for the case a<0, b>0 is

(157) $$Q\left(\xi \right)=-\sqrt[{3}]{10\nu_{1}b^{2}}{\left(-\frac{\frac{\mu_{3}\sqrt{-\nu_{1}\mu_{3}}}{8\sqrt{10}\nu_{1}\mu_{4}}\exp\left[3\frac{\mu_{3}\sqrt{-\nu_{1}\mu_{3}}}{8\sqrt{10}\nu_{1}\mu_{4}}\left(\xi +\xi_{0}\right)\right]}{1+b\exp\left[3\frac{\mu_{3}\sqrt{-\nu_{1}\mu_{3}}}{8\sqrt{10}\nu_{1}\mu_{4}}\left(\xi +\xi_{0}\right)\right]}\right)}^{2/3}-\frac{\mu_{3}}{4\mu_{4}}.$$

for the case b>0 and a<0.

Next, we can set N=2,M=5. The equation that has to be solved is

(158) $$\nu_{1}\frac{d^{2}Q}{d\xi^{2}}+\nu_{2}\frac{dQ}{d\xi }+\mu_{5}Q^{5}+\mu_{4}Q^{4}+\mu_{3}Q^{3}+\mu_{2}Q^{2}+\mu_{1}Q=0.$$

The SEsM leads to the conclusion that this equation has solutions of the kind $Q\left(\xi \right)=\sum_{i=0}^{L}a_{i}{\left[\phi \left(\xi \right)\right]}^{i},\;\frac{d\phi }{d\xi }=b\phi^{\left(1+2L\right)}+a\phi$. For the case L=1 and for a>0, b<0, the solution is [478]

(159) $$Q\left(\xi \right)=a_{1}\sqrt{\frac{Q_{1}}{Q_{2}}}+a_{0},$$

where

$$\begin{array}{c} Q_{1}=-\frac{4\nu_{1}ba_{0}^{2}+\nu_{2}a_{1}^{2}+\sqrt{4\nu_{1}a_{0}b\left(\nu_{1}ba_{0}^{3}+2a_{0}\nu_{2}a_{1}^{2}\right)+\nu_{2}a_{1}\left(\nu_{2}a_{1}^{3}+4\nu_{1}ba_{0}^{3}\right)}}{2\nu_{1}a_{1}^{2}}\times \\ \exp\left[-2\left(\frac{4\nu_{1}ba_{0}^{2}+\nu_{2}a_{1}^{2}+\sqrt{4\nu_{1}a_{0}b\left(\nu_{1}ba_{0}^{3}+2a_{0}\nu_{2}a_{1}^{2}\right)+\nu_{2}a_{1}\left(\nu_{2}a_{1}^{3}+4\nu_{1}ba_{0}^{3}\right)}}{2\nu_{1}a_{1}^{2}}\right)\left(\xi +\xi_{0}\right)\right]. \end{array}$$

$$Q_{2}=1-$$

$$b\exp\left[-2\left(\frac{4\nu_{1}ba_{0}^{2}+\nu_{2}a_{1}^{2}+\sqrt{4\nu_{1}a_{0}b\left(\nu_{1}ba_{0}^{3}+2a_{0}\nu_{2}a_{1}^{2}\right)+\nu_{2}a_{1}\left(\nu_{2}a_{1}^{3}+4\nu_{1}ba_{0}^{3}\right)}}{2\nu_{1}a_{1}^{2}}\right)\left(\xi +\xi_{0}\right)\right].$$

The solutions above are for the use of the equation of Bernoulli as the simple equation in the SEsM. One can also use other equations as simple equations. One example is the equation of Riccati. Numerous solutions of the discussed equations obtained based on the application of the SEsM based on the Riccati equation as the simple equation can be seen in [478].

The SEsM has been applied to obtain exact solutions of more complicated classes of equations. One such class is connected to modeling PDEs from ecology and population dynamics [480]

(160) $$\begin{array}{c} \sum_{p=1}^{N_{1}}A_{p}\left(Q\right)\frac{\partial^{p}Q}{\partial t^{p}}+\sum_{r=2}^{N_{2}}B_{r}\left(Q\right){\left(\frac{\partial Q}{\partial t}\right)}^{r}+\sum_{s=1}^{N_{3}}C_{s}\left(Q\right)\frac{\partial^{s}Q}{\partial x^{s}}+\sum_{u=2}^{N_{4}}D_{u}\left(Q\right){\left(\frac{\partial Q}{\partial x}\right)}^{u}+F\left(Q\right)=0. \end{array}$$

$A_{p}\left(Q\right)$, $B_{r}\left(Q\right)$, $C_{s}\left(Q\right)$, $D_{u}\left(Q\right)$, and F(Q) are polynomials of *Q*. Specific cases of (160) have been obtained as model equations in the theory of the migration of populations [476,477]. We search for traveling wave solutions Q(x,t)=Q(ξ)=Q(x−vt). This transforms (160) into

(161) $$\sum_{p=0}^{n_{1}}{\bar{A}}_{p}\left(Q\right)\frac{d^{p}Q}{d\xi^{p}}+\sum_{r=2}^{n_{2}}{\bar{B}}_{r}\left(Q\right){\left(\frac{dQ}{d\xi }\right)}^{r}+F\left(Q\right)=0.$$

In (161), $n_{1}=\max\left(N_{1},N_{3}\right)$, $n_{2}=\max\left(N_{2},N_{4}\right)$ and ${\bar{A}}_{p}\left(Q\right)=A_{p}\left(Q\right){\left(-v\right)}^{p}+C_{p}\left(Q\right),{\bar{B}}_{r}\left(Q\right)=B_{r}\left(Q\right){\left(-v\right)}^{r}+D_{r}\left(Q\right)$. ${\bar{A}}_{p}$, ${\bar{B}}_{r}$, and *F* are assumed as follows: ${\bar{A}}_{p}\left(Q\right)=\sum_{i=0}^{M_{1p}}q_{i}Q^{i},$ ${\bar{B}}_{r}\left(Q\right)=\sum_{i=0}^{M_{2r}}q^{*}Q^{i},F\left(Q\right)=\sum_{i=0}^{M}\bar{q}Q^{i}$. Here, $q_{i}$, $q_{i}^{*}$, and ${\bar{q}}_{i}$ are coefficients. We discuss obtaining the balance equations for several specific cases below. The corresponding exact solutions can be seen in [480].

We discuss the case of the application of the SEsM for obtaining the exact solution of (161) when the simple equation is the equation of Bernoulli and the solution is searched in the form

(162) $$Q\left(\xi \right)=\sum_{i=0}^{L}a_{i}{\left[\phi \left(\xi \right)\right]}^{i}.$$

ϕ(ξ) is a solution of the equation of Bernoulli, which is used here as the simple equation. Thus, we fix the form of the composite function for the solution and we fix the simple equation. Next, we have to go to Step 3 of the SEsM to obtain the balance equations. In order to obtain the balance, we have to estimate the maximum powers of the term of the solved equation for the case when the equation of Bernoulli is used as the simple equation. We name the maximum powers ${\mathrm{BL}}_{1p}$, ${\mathrm{BL}}_{2r}$, and ${\mathrm{BL}}_{3}$. The estimations are as follows:

$$\mathrm{Term}\;{\bar{A}}_{p}\left(Q\right)\frac{d^{p}Q}{d\xi^{p}}\to {\mathrm{BL}}_{1p}=LM_{1p}+L+p\left(k-1\right),\;p=0,1,\ldots ,n_{1}$$

$LM_{1p}$ is the maximum power of ϕ(ξ) arising from the polynomial ${\bar{A}}_{p}\left(Q\right)$.

$$\mathrm{Term}\;{\bar{B}}_{r}\left(Q\right){\left(\frac{dQ}{d\xi }\right)}^{r}\to {\mathrm{BL}}_{2r}=LM_{2r}+r\left(L+k-1\right),\;r=2,\ldots ,n_{1}$$

$LM_{2r}$ is the maximum power of ϕ(ξ) arising from the polynomial ${\bar{B}}_{r}\left(Q\right)$.

$$\mathrm{Term}\;F\left(Q\right)\to {\mathrm{BL}}_{3}=LM$$

We denote as max(${\mathrm{BL}}_{1p}$) the largest value among all ${\mathrm{BL}}_{1p}$ and with max(${\mathrm{BL}}_{2r}$), the largest value among all ${\mathrm{BL}}_{2r}$. The theoretically possible balance equations are

(163) $$\max\left({\mathrm{BL}}_{1p}\right)=\max\left({\mathrm{BL}}_{2r}\right);\;\max\left({\mathrm{BL}}_{1p}\right)=LM;\;\max\left({\mathrm{BL}}_{2r}\right)=LM.$$

Often, one of these three terms is smaller than the other two.

Several examples of the determination of the balance equations are below. For the equation

(164) $$\frac{\partial Q}{\partial t}=D\frac{\partial^{2}Q}{\partial x^{2}}+\frac{d^{2}\Psi }{dQ^{2}}{\left(\frac{\partial Q}{\partial x}\right)}^{2}+\frac{d\Psi }{dQ}\frac{\partial^{2}Q}{\partial x^{2}},$$

where *D* is the constant coefficient of diffusion, Ψ(Q) is a polynomial of *Q* as follows $\Psi \left(Q\right)=\sum_{m_{1}=0}^{M_{1}}a_{m_{1}}Q^{m_{1}}$. The largest powers of ϕ(ξ) are

$${\mathrm{BL}}_{11}=L+k-1;\;{\mathrm{BL}}_{12}=LM_{1}+2\left(k-1\right);\;{\mathrm{BL}}_{22}=LM_{1}+2\left(k-1\right).$$

Equation (164) has to be a nonlinear PDE. Then, $M_{1}>2$. This leads to ${\mathrm{BL}}_{12}>{\mathrm{BL}}_{11}$ and ${\mathrm{BL}}_{22}>{\mathrm{BL}}_{11}$. The balance equation becomes

(165) $${\mathrm{BL}}_{12}={\mathrm{BL}}_{22}.$$

The next example is the balance equation for the reaction–diffusion equation with a diffusion coefficient that depends on population density

(166) $$\frac{\partial Q}{\partial t}=\frac{dD}{dQ}{\left(\frac{\partial Q}{\partial x}\right)}^{2}+D\left(Q\right)\frac{\partial^{2}Q}{\partial x^{2}}+F\left(Q\right)=0,$$

where we assume $D\left(Q\right)=\sum_{m_{1}=0}^{M_{1}}a_{m_{1}}Q^{m_{1}};\;F\left(Q\right)=\sum_{m=0}^{M}b_{m}Q^{m}$. For the case of the use of the Bernoulli equation as the simple equation, the largest powers of ϕ(ξ) arising from the four terms of (166) are ${\mathrm{BL}}_{11}=L+k-1;\;{\mathrm{BL}}_{12}=LM_{1}+L+2\left(k-1\right)$, ${\mathrm{BL}}_{22}=L\left(M_{1}-1\right)+2\left(L+k-1\right);\;{\mathrm{BL}}_{3}=LM$. ${\mathrm{BL}}_{11}$ is smaller than ${\mathrm{BL}}_{12}={\mathrm{BL}}_{22}$. Two possibilities remain. If *M* is such that ${\mathrm{BL}}_{3}$ is smaller than ${\mathrm{BL}}_{12}$, then the balance equation is ${\mathrm{BL}}_{12}={\mathrm{BL}}_{22},$ and no requirements are imposed on *L* and $M_{1}$. The second possibility is to balance ${\mathrm{BL}}_{12}$ and ${\mathrm{BL}}_{3}$. Thus, ${\mathrm{BL}}_{12}={\mathrm{BL}}_{3}\to L\left(M_{1}+1\right)+2\left(k-1\right)=LM$.

The balance equations for the other differential equations of the studied class, as well as the corresponding exact solutions, can be seen in [480].

A more complicated example is connected to the extended Camassa–Holm equation [484]. The Camassa–Holm equation is a model equation for the x− component *u* of the fluid velocity at a certain depth $z_{0}$ below the fluid surface. The Camassa–Holm equation was introduced as a completely integrable bi-Hamiltonian dispersive shallow water equation [506]. The Camassa–Holm equation is [484,507]

(167) $$\frac{\partial U}{\partial T}+2\kappa \frac{\partial U}{\partial X}+3\epsilon U\frac{\partial U}{\partial X}-\epsilon \delta^{2}\frac{\partial^{3}U}{\partial X^{2}\partial T}=\epsilon^{2}\delta^{2}\left(2\frac{\partial U}{\partial X}\frac{\partial^{2}U}{\partial X^{2}}+U\frac{\partial^{3}U}{\partial X^{3}}\right).$$

ϵ and δ are the amplitude parameter and the shallowness parameter and *U* is a quantity connected to the height of the wave η over the water surface.

(168) $$\eta ∼\sqrt{\frac{5}{3}}\left(U+\frac{1}{4}\sqrt{\frac{5}{3}}\epsilon U^{2}-\frac{1}{5}\epsilon \delta^{2}\frac{\partial^{2}U}{\partial X^{2}}\right).$$

Below, we present an exact solution of the generalized Camassa–Holm equation [484]

(169) $$\begin{array}{c} \frac{\partial U}{\partial T}+p_{1}\frac{\partial U}{\partial X}-p_{2}\frac{\partial^{3}U}{\partial X^{2}\partial T}-p_{3}\frac{\partial^{3}}{\partial X^{3}}f\left(U\right)+p_{4}\frac{\partial }{\partial X}\left[\frac{1}{2}g\left(U\right)+\frac{p_{5}}{2}\frac{d^{2}f}{dU^{2}}{\left(\frac{\partial U}{\partial X}\right)}^{2}\right]=0. \end{array}$$

$p_{1};p_{2};p_{3};p_{4};p_{5}$ are parameters. Specific cases of (169) are as below. For $f\left(U\right)=\frac{1}{2}U^{2}$; $g\left(U\right)=C+3U^{2}$ (*C* is a constant) and $p_{3}=p_{4}p_{5}$, one obtains

(170) $$\frac{\partial U}{\partial T}+p_{1}\frac{\partial U}{\partial X}-p_{2}\frac{\partial^{3}U}{\partial X^{2}\partial T}+3p_{4}U\frac{\partial U}{\partial X}-2p_{3}\frac{\partial U}{\partial X}\frac{\partial^{2}U}{\partial X^{2}}-p_{3}U\frac{\partial^{3}U}{\partial X^{3}}=0.$$

Equation (169) contains the Camassa–Holm equation as a particular case when $p_{2}=2\kappa$; $p_{4}=\epsilon$; $p_{2}=\epsilon \delta^{2}$; $p_{3}=\epsilon^{2}\delta^{2}$. In general, in Equation (169), f(U) and g(U) can be arbitrary polynomials of *U*.

We skip Step 1 of the SEsM (no transformation of the nonlinearity). We search for traveling wave solutions and introduce the coordinate ξ=X−vT, where *v* is the velocity of the traveling wave. In Step 2 of the SEsM, we fix the composite function for the solution to be a power series of the solution of the simple equation. In Step 3 of the SEsM, we have to determine the balance equations for the corresponding simple equations. For the case when the simple equation is the equation of Bernoulli, the balance equation is

(171) $$\nu_{1}=\frac{2(k-1)}{J-I}.$$

where k>1 and J>I>1. For I>1, another balance equation is possible. It is

(172) $$I\nu_{1}+3\left(k-1\right)=I\nu_{1}+3\left(k-1\right),$$

when $\left(J-I\right)\nu_{1}<2\left(k-1\right)$. This balance is possible because of the existence of two terms in (169), which lead to the maximum power $I\nu_{1}+3\left(k-1\right)$ of the terms of the resulting polynomial, and these powers are maximum powers for the entire polynomial when $\left(J-I\right)\nu_{1}<2\left(k-1\right)$.

A third balance equation is possible in the case of the use of the Bernoulli equation as the simple equation. This balance is when I=1. The corresponding balance equation is

(173) $$\nu_{1}=\frac{2(k-1)}{J-1}.$$

Another possibility for the simple equation is the equation of Riccati (136). The solution of Equation (169) is, again, of the kind (132). The possible balance equations in this case are

(174) $$\nu_{1}=I;\;J<\nu_{1}+2;\;J<I+2,$$

(175) $$\nu_{1}+2=J;\;I<\nu_{1};\;I+2<J,$$

(176) $$J=I+2;\;\nu_{1}<I;\;\nu_{1}+2<J.$$

The possible balance equations for the case when the extended tanh-equation is used as the simplest equation are the same as for the case of the Riccati equation.

We return to the case of the use of the SEsM with the equation of Bernoulli as the simple equation. The values of parameters in the balance equation, Equation (171), are set to J=3, I=2, k=2. Then, from (171), $\nu_{1}=2$. The equation we solve is

(177) $$\begin{array}{c} \frac{\partial U}{\partial T}+p_{1}\frac{\partial U}{\partial X}-p_{2}\frac{\partial^{3}U}{\partial X^{2}\partial T}-p_{3}\frac{\partial^{3}}{\partial X^{3}}\left(q_{0}+q_{1}U+q_{2}U^{2}\right)+\\ p_{4}\frac{\partial }{\partial X}\left[\frac{1}{2}\left(r_{0}+r_{1}U+r_{2}U^{2}+r_{3}U^{3}\right)+p_{5}q_{2}{\left(\frac{\partial U}{\partial X}\right)}^{2}\right]=0. \end{array}$$

The solution of Equation (170) is searched in the form

(178) $$U\left(\xi \right)=\theta_{0}+\theta_{1}V\left(\xi \right)+\theta_{2}V{\left(\xi \right)}^{2}.$$

V(ξ) is the solution of the equation of Bernoulli for k=2. Substituting (178) in (170), we obtain a system of seven nonlinear relationships between the parameters of the solved equation and the parameters of the solution (178). Step 4 of the SEsM is to solve this system of algebraic equations. One nontrivial solution is

(179) $$\begin{array}{ccc} \theta_{2} & = & 8\frac{q_{2}ac\left(5p_{3}-p_{4}p_{5}\right)}{p_{4}r_{3}};\;\theta_{1}=8\frac{q_{2}c^{2}\left(5p_{3}-p_{4}p_{5}\right)}{p_{4}r_{3}};\\ \theta_{0} & = & \frac{\Theta_{0}}{6q_{2}p_{4}r_{3}\left(4p_{3}-p_{4}p_{5}\right)};\;r_{1}=\frac{R_{1}}{12p_{2}p_{4}r_{3}\left(4p_{3}-p_{4}p_{5}\right)};\\ \Theta_{0} & = & -36q_{2}^{2}a^{2}p_{4}p_{5}p_{3}+4q_{2}^{2}a^{2}p_{4}^{2}p_{5}^{2}-3p_{2}vp_{4}r_{3}+3p_{3}q_{1}p_{4}r_{3}+80q_{2}^{2}a^{2}p_{3}^{2}-\\ & & 10q_{2}p_{3}r_{2}p_{4}+2q_{2}p_{4}^{2}p_{5}r_{2};\\ R_{1} & = & -(1520q_{2}^{4}a^{4}p_{4}^{2}p_{5}^{2}p_{3}^{2}-256q_{2}^{4}a^{4}p_{4}^{3}p_{5}^{3}p_{3}-4q_{2}^{2}p_{4}^{4}p_{5}^{2}r_{2}^{2}+32q_{2}^{2}p_{3}r_{2}^{2}p_{4}^{3}p_{5}-\\ & & 60q_{2}^{2}p_{3}^{2}r_{2}^{2}p_{4}^{2}+3840q_{2}^{4}a^{4}p_{3}^{4}-12p_{3}^{2}q_{1}p_{4}^{2}r_{3}q_{2}r_{2}+12p_{2}vp_{4}^{2}r_{3}q_{2}p_{3}r_{2}-\\ & & 18p_{2}vp_{4}^{2}r_{3}^{2}p_{3}q_{1}+9p_{3}^{2}q_{1}^{2}p_{4}^{2}r_{3}^{2}+16q_{2}^{4}a^{4}p_{4}^{4}p_{5}^{4}+9p_{2}^{2}v^{2}p_{4}^{2}r_{3}^{2}-\\ & & 3968q_{2}^{4}a^{4}p_{4}p_{5}p_{3}^{3}-24vq_{2}^{2}r_{3}p_{4}^{3}p_{5}^{2}+192vq_{2}^{2}r_{3}p_{4}^{2}p_{3}p_{5}-384vq_{2}^{2}r_{3}p_{4}p_{3}^{2}-\\ & & 192p_{1}q_{2}^{2}r_{3}p_{4}^{2}p_{3}p_{5}+384p_{1}q_{2}^{2}r_{3}p_{4}p_{3}^{2}+24p_{1}q_{2}^{2}r_{3}p_{4}^{3}p_{5}^{2}). \end{array}$$

This leads to the following solution of Equation (177):

(180) $$\begin{array}{c} U\left(\xi \right)=\frac{\Theta_{0}}{6q_{2}p_{4}r_{3}\left(4p_{3}-p_{4}p_{5}\right)}+8\frac{q_{2}c^{2}\left(5p_{3}-p_{4}p_{5}\right)}{p_{4}r_{3}}\left\{\frac{a\exp[a\left(\xi +\xi_{0}\right)]}{1-c\exp[a\left(\xi +\xi_{0}\right)]}\right\}+\\ 8\frac{q_{2}ac\left(5p_{3}-p_{4}p_{5}\right)}{p_{4}r_{3}}{\left\{\frac{a\exp[a\left(\xi +\xi_{0}\right)]}{1-c\exp[a\left(\xi +\xi_{0}\right)]}\right\}}^{2}, \end{array}$$

for c<0 and a>0. For c>0 and a<0, the solution is

(181) $$\begin{array}{c} U\left(\xi \right)=\frac{\Theta_{0}}{6q_{2}p_{4}r_{3}\left(4p_{3}-p_{4}p_{5}\right)}-8\frac{q_{2}c^{2}\left(5p_{3}-p_{4}p_{5}\right)}{p_{4}r_{3}}\left\{\frac{a\exp[a\left(\xi +\xi_{0}\right)]}{1+c\exp[a\left(\xi +\xi_{0}\right)]}\right\}+\\ 8\frac{q_{2}ac\left(5p_{3}-p_{4}p_{5}\right)}{p_{4}r_{3}}{\left\{\frac{a\exp[a\left(\xi +\xi_{0}\right)]}{1+c\exp[a\left(\xi +\xi_{0}\right)]}\right\}}^{2}. \end{array}$$

Another possibility is to use the equation of Riccati (136) as the simple equation. We use (174) for the balance equation and set the parameters to J=1; $\nu_{1}=2$; I=2. The equation that is to be solved is

(182) $$\begin{array}{c} \frac{\partial U}{\partial T}+p_{1}\frac{\partial U}{\partial X}-p_{2}\frac{\partial^{3}U}{\partial X^{2}\partial T}-p_{3}\frac{\partial^{3}}{\partial X^{3}}\left(q_{0}+q_{1}U+q_{2}U^{2}\right)+p_{4}\frac{\partial }{\partial X}\left[\frac{1}{2}\left(r_{0}+r_{1}U\right)+p_{5}q_{2}{\left(\frac{\partial U}{\partial X}\right)}^{2}\right]=0. \end{array}$$

In Step 4 of the SEsM, we obtain a nonlinear algebraic system of eight relationships between the parameters of the solved equation and the parameters of the solution. A nontrivial solution of this system is

(183) $$\begin{array}{ccc} \theta_{2} & = & -15\frac{a^{2}\left(-p_{4}r_{1}+2v-2p_{1}\right)}{q_{2}p_{4}p_{4}\left(-8c^{2}da+16a^{2}d^{2}+c^{4}\right)},\\ \theta_{1} & = & -15\frac{ac(-p_{4}r_{1}+2v-2p_{1})}{q_{2}p_{4}p_{4}\left(-8c^{2}da+16a^{2}d^{2}+c^{4}\right)},\\ \theta_{0} & = & \frac{\Theta_{0}}{q_{2}p_{4}p_{5}\left(-8c^{2}da+16a^{2}d^{2}+c^{4}\right)},\\ \Theta_{0} & = & \frac{1}{4}(40adp_{4}r_{1}-80adv+80adp_{1}+5c^{2}p_{4}r_{1}-10c^{2}v+10c^{2}p_{1}+\\ & & 16p_{4}p_{5}q_{1}c^{2}da-32p_{4}p_{5}q_{1}a^{2}d^{2}-2p_{4}p_{5}q_{1}c^{4}-80p_{2}vc^{2}da+160p_{2}va^{2}d^{2}+\\ & & 10p_{2}vc^{4}). \end{array}$$

The corresponding solution of Equation (182) based on the solution (137) of the Riccati equation is

(184) $$\begin{array}{ccc} U(\xi ) & = & \frac{\Theta_{0}}{q_{2}p_{4}p_{5}\left(-8c^{2}da+16a^{2}d^{2}+c^{4}\right)}+\\ & & 15\frac{ac(-p_{4}r_{1}+2v-2p_{1})}{q_{2}p_{4}p_{4}\left(-8c^{2}da+16a^{2}d^{2}+c^{4}\right)}\left[\frac{c}{2a}+\frac{\theta }{2a}\tanh\left(\frac{\theta (\xi +\xi_{0})}{2}\right)\right]-\\ & & 15\frac{a^{2}\left(-p_{4}r_{1}+2v-2p_{1}\right)}{q_{2}p_{4}p_{4}\left(-8c^{2}da+16a^{2}d^{2}+c^{4}\right)}{\left[\frac{c}{2a}+\frac{\theta }{2a}\tanh\left(\frac{\theta (\xi +\xi_{0})}{2}\right)\right]}^{2}. \end{array}$$

In (184), $\theta^{2}=c^{2}-4ad>0$.

Finally, we can use the extended tanh-equation, Equation (138), as the simple equation. From the corresponding balance equation, we choose the parameters I=J=2 and $\nu_{1}=2$. The equation to be solved is

(185) $$\begin{array}{c} \frac{\partial U}{\partial T}+p_{1}\frac{\partial U}{\partial X}-p_{2}\frac{\partial^{3}U}{\partial X^{2}\partial T}-p_{3}\frac{\partial^{3}}{\partial X^{3}}\left(q_{0}+q_{1}U+q_{2}U^{2}\right)+\\ p_{4}\frac{\partial }{\partial X}\left[\frac{1}{2}\left(r_{0}+r_{1}U+r_{2}U^{2}\right)+p_{5}q_{2}{\left(\frac{\partial U}{\partial X}\right)}^{2}\right]=0. \end{array}$$

In Step 4 of the SEsM, one obtains a system of nonlinear algebraic equations that contains eight relationships. A nontrivial solution of this system is

(186) $$\begin{array}{ccc} \theta_{2} & = & 60{\bar{a}}^{4}\frac{\left(-2vp_{5}q_{2}+2p_{1}p_{5}q_{2}+r_{1}p_{4}p_{5}q_{2}+5r_{2}p_{2}v-r_{2}p_{4}p_{5}q_{1}\right)}{p_{4}\left(64p_{5}^{2}q_{2}^{2}{\bar{d}}^{4}{\bar{a}}^{2}-25r_{2}^{2}\right)},\\ \theta_{1} & = & 0,\\ \theta_{0} & = & \frac{\Theta_{0}}{p_{4}\left(-25r_{2}^{2}+64p_{5}^{2}q_{2}^{2}{\bar{d}}^{4}{\bar{a}}^{4}\right)},\\ \Theta_{0} & = & \frac{1}{2}(160p_{5}q_{2}{\bar{a}}^{2}{\bar{d}}^{2}v-160p_{5}q_{2}{\bar{a}}^{2}{\bar{d}}^{2}p_{1}-80p_{5}q_{2}{\bar{a}}^{2}{\bar{d}}^{2}r_{1}p_{4}-400{\bar{a}}^{2}{\bar{d}}^{2}r_{2}p_{2}v+\\ & & 80p_{5}{\bar{a}}^{2}{\bar{d}}^{2}r_{2}p_{4}q_{1}+320p_{2}vp_{5}q_{2}{\bar{d}}^{4}{\bar{a}}^{4}-50r_{2}v+50r_{2}p_{1}+25r_{2}r_{1}p_{4}-\\ & & 64p_{4}p_{5}^{2}q_{1}q_{2}{\bar{d}}^{4}{\bar{a}}^{4})\\ p_{3} & = & \frac{1}{5}p_{4}p_{5}. \end{array}$$

The corresponding solution of the solved equation, Equation (185), is

(187) $$\begin{array}{ccc} U(\xi ) & = & \frac{\Theta_{0}}{p_{4}\left(-25r_{2}^{2}+64p_{5}^{2}q_{2}^{2}{\bar{d}}^{4}{\bar{a}}^{4}\right)}+\\ & & 60{\bar{a}}^{2}{\bar{d}}^{2}\frac{\left(-2vp_{5}q_{2}+2p_{1}p_{5}q_{2}+r_{1}p_{4}p_{5}q_{2}+5r_{2}p_{2}v-r_{2}p_{4}p_{5}q_{1}\right)}{p_{4}\left(64p_{5}^{2}q_{2}^{2}{\bar{d}}^{4}{\bar{a}}^{2}-25r_{2}^{2}\right)}\tanh^{2}\left[\bar{a}\bar{d}\left(\xi +\xi_{0}\right)\right]. \end{array}$$

The next example we consider is connected to the generalized Swift–Hohenberg equation. The 1+1-dimensional version is discussed:

(188) $$\frac{\partial u}{\partial t}=ru-{\left(1+\nabla^{2}\right)}^{2}u+N\left(u\right).$$

Here, *r* is a parameter and N(u) is a polynomial nonlinearity $N\left(u\right)\to \sum_{s=0}^{s^{*}}c_{s}^{*}u^{s}$, where $c_{s}^{*}$, s=0,1,2,… are parameters. Traveling wave solutions are searched. We set $c_{0}=c_{0}^{*}$; $c_{1}=c_{1}^{*}+r-1$; …, $c_{s}=c_{s}^{*}$, and the solved equation becomes

(189) $$\frac{\partial u}{\partial t}+2\frac{\partial^{2}u}{\partial x^{2}}+\frac{\partial u^{4}}{\partial x^{4}}=\sum_{s=0}^{s^{*}}c_{s}u^{s}.$$

The balance equation that is obtained in Step 3 of the SEsM is

(190) $$\eta =\frac{4(\beta -1)}{s^{*}-1}.$$

We consider the specific case $s^{*}=5$. The corresponding Swift–Hohenberg equation becomes

(191) $$\begin{array}{c} \frac{\partial u}{\partial t}+2\frac{\partial^{2}u}{\partial x^{2}}+\frac{\partial^{4}u}{\partial x^{4}}=c_{0}+c_{1}u+c_{2}u^{2}+c_{3}u^{3}+c_{4}u^{4}+c_{5}u^{5}. \end{array}$$

The balance equation is

(192) η=β−1.

For β=2, η=1. β=2 means that we use the equation of Riccati (136) or the extended tanh-Equation (138) as the simple equation. For the case of the use of the extended tanh-equation as the simple equation, in Step 4 of the SEsM, one obtains the system of nonlinear algebraic relationships.

(193) $$\begin{array}{ccc} 24a^{4}-c_{5}\theta_{1}^{4} & = & 0,\\ c_{4}+5c_{5}\theta_{0} & = & 0,\\ 40a^{3}c+4a^{2}-c_{3}\theta_{1}^{2}-10c_{5}\theta_{0}^{2}\theta_{1}^{2}-4c_{4}\theta_{0}\theta_{1}^{2} & = & 0,\\ -va-c_{2}\theta_{1}-3c_{3}\theta_{0}\theta_{1}-10c_{5}\theta_{0}^{3}\theta_{1}-6c_{4}\theta_{0}^{2}\theta_{1} & = & 0,\\ -2c_{2}\theta_{0}+4ca+16a^{2}c^{2}-5c_{5}\theta_{0}^{4}-3c_{3}\theta_{0}^{2}-4c_{4}\theta_{0}^{3}-c_{1} & = & 0,\\ -c_{0}-c_{1}\theta_{0}-v\theta_{1}c-c_{2}\theta_{0}^{2}-c_{4}\theta_{0}^{4}-c_{5}\theta_{0}^{5}-c_{3}\theta_{0}^{3} & = & 0. \end{array}$$

A nontrivial solution of this system is

(194) $$\begin{array}{ccc} c_{5} & = & \frac{24a^{4}}{\theta_{1}^{4}},\\ c_{4} & = & -\frac{120a^{4}\theta_{0}}{\theta_{1}^{4}},\\ c_{3} & = & 4a^{2}\frac{10\theta_{1}^{2}ac+\theta_{1}^{2}+60a^{2}\theta_{0}^{2}}{\theta_{1}^{4}},\\ c_{2} & = & -a\frac{v\theta_{1}^{3}+120a^{2}\theta_{0}\theta_{1}^{2}c+12a\theta_{0}\theta_{1}^{2}+240a^{3}\theta_{0}^{3}}{\theta_{1}^{4}},\\ c_{1} & = & 2a\frac{\theta_{0}v\theta_{1}^{3}+60a^{2}\theta_{0}^{2}\theta_{1}^{2}c+6a\theta_{0}^{2}\theta_{1}^{2}+60a^{3}\theta_{0}^{4}+2\theta_{1}^{4}c+8\theta_{1}^{4}ac^{2}}{\theta_{1}^{4}},\\ c_{0} & = & -\frac{24a^{4}\theta_{0}^{5}+a\theta_{0}^{2}v\theta_{1}^{3}+40a^{3}\theta_{0}^{3}\theta_{1}^{2}c+4a^{2}\theta_{0}^{3}\theta_{1}^{2}+4a\theta_{0}\theta_{1}^{4}c+16a^{2}\theta_{0}\theta_{1}^{4}c^{2}+v\theta_{1}^{5}c}{\theta_{1}^{4}}. \end{array}$$

The corresponding solution of (191) is

(195) $$u\left(\xi \right)=\theta_{0}+\theta_{1}\frac{\bar{c}}{\bar{a}}\tanh\left[\bar{a}\;\bar{c}\left(\xi +\xi_{0}\right)\right],$$

where $a=-{\bar{a}}^{2}$ and $c={\bar{c}}^{2}$.

For the case β=3, one obtains from (192) η=2. The simple equation is

(196) $$\frac{dV}{d\xi }=\gamma_{0}+\gamma_{1}V+\gamma_{2}V^{2}+\gamma_{3}V^{3},$$

which for $\gamma_{0}=\gamma_{2}=0$ and $\gamma_{1}=a$, as well as $\gamma_{3}=b$, is reduced to the equation of Bernoulli (134). A nontrivial solution of the corresponding system of nonlinear algebraic relationships obtained in Step 4 of the SEsM is

(197) $$\begin{array}{ccc} \theta_{1} & = & 0,\\ c_{5} & = & 384\frac{b^{4}}{\theta_{2}^{4}},\\ c_{4} & = & 960\frac{b^{3}\left(-2b\theta_{0}+\theta_{2}a\right)}{\theta_{2}^{4}},\\ c_{3} & = & 16\frac{b^{2}\left(240b^{2}\theta_{0}^{2}-240b\theta_{0}\theta_{2}a+50\theta_{2}^{2}a^{2}+\theta_{2}^{2}\right)}{\theta_{2}^{4}},\\ c_{2} & = & \frac{2b}{\theta_{2}^{4}}(-v\theta_{2}^{3}+120\theta_{2}^{3}a^{3}+12\theta_{2}^{3}a-1920b^{3}\theta_{0}^{3}+2880b^{2}\theta_{0}^{2}\theta_{2}a-\\ & & 1200b\theta_{0}\theta_{2}^{2}a^{2}-24b\theta_{0}\theta_{2}^{2}),\\ c_{1} & = & \frac{2}{\theta_{2}^{4}}(960b^{4}\theta_{0}^{4}-1920b^{3}\theta_{0}^{3}\theta_{2}a+1200b^{2}\theta_{0}^{2}\theta_{2}^{2}a^{2}+24b^{2}\theta_{0}^{2}\theta_{2}^{2}-va\theta_{2}^{4}+8a^{4}\theta_{2}^{4}+\\ & & 2b\theta_{0}v\theta_{2}^{3}-240b\theta_{0}\theta_{2}^{3}a^{3}-24b\theta_{0}\theta_{2}^{3}a+4a^{2}\theta_{2}^{4}),\\ c_{0} & = & -\frac{2\theta_{0}}{\theta_{2}^{4}}(192b^{4}\theta_{0}^{4}-480b^{3}\theta_{0}^{3}\theta_{2}a+400b^{2}\theta_{0}^{2}\theta_{2}^{2}a^{2}+\\ & & 8b^{2}\theta_{0}^{2}\theta_{2}^{2}-va\theta_{2}^{4}+8a^{4}\theta_{2}^{4}+b\theta_{0}v\theta_{2}^{3}-120b\theta_{0}\theta_{2}^{3}a^{3}-12b\theta_{0}\theta_{2}^{3}a+4a^{2}\theta_{2}^{4}). \end{array}$$

The solution of the Swift–Hohenberg Equation (191) is

(198) $$u\left(\xi \right)=\theta_{0}+\theta_{2}\frac{a\exp[2a\left(\xi +\xi_{0}\right)]}{1-b\exp[2a\left(\xi +\xi_{0}\right)]},$$

for the case b<0, a>0 and

(199) $$u\left(\xi \right)=\theta_{0}-\theta_{2}\frac{a\exp[2a\left(\xi +\xi_{0}\right)]}{1+b\exp[2a\left(\xi +\xi_{0}\right)]},$$

for the case b>0, a<0. $\theta_{0}$ and $\theta_{2}$ are free parameters.

The next example is connected to the generalized Rayleigh equation

(200) $$\frac{\partial^{2}u}{\partial t^{2}}-\frac{\partial^{2}u}{\partial x^{2}}+F\left(\frac{\partial u}{\partial t}\right)+G\left(u\right)=0.$$

*F* and *G* are the functions of the corresponding terms. For u=u(t), G(u)=u, one obtains the Rayleigh equation. For $F\left(\frac{du}{dt}\right)=-\epsilon \left(\frac{du}{dt}-{\left(\frac{du}{dt}\right)}^{2}\right)$, one obtains the specific case of the Rayleigh equation

(201) $$\frac{d^{2}y}{dt^{2}}-\epsilon \left(\frac{du}{dt}-{\left(\frac{du}{dt}\right)}^{2}\right)+u=0.$$

Equation (201) occurs in the theory of sound.

We consider the case where F and G are polynomials

(202) $$F\left(\frac{\partial u}{\partial t}\right)=\sum_{j=0}^{m}a_{j}{\left(\frac{\partial u}{\partial t}\right)}^{j};\;G\left(u\right)=-\sum_{s=0}^{s^{*}}c_{s}u^{s}.$$

Traveling wave solutions u(x,t)=u(ξ)=u(x−vt) are searched for the solved equation

(203) $$\frac{\partial^{2}u}{\partial t^{2}}+\sum_{j=0}^{m}a_{j}{\left(\frac{\partial u}{\partial t}\right)}^{j}-\frac{\partial^{2}u}{\partial x^{2}}=\sum_{s=0}^{s^{*}}c_{s}u^{s}.$$

The balance equation that occurs in Step 3 of the SEsM is

(204) $$\eta =\frac{m(\beta -1)}{s^{*}-m}.$$

For the case m=2, we solve the equation

(205) $$\frac{\partial^{2}u}{\partial t^{2}}+\sum_{j=0}^{2}a_{j}{\left(\frac{\partial u}{\partial t}\right)}^{j}-\frac{\partial^{2}u}{\partial x^{2}}=\sum_{s=0}^{s^{*}}c_{s}u^{s},$$

and the balance equation is

(206) $$\eta =\frac{2(\beta -1)}{s^{*}-2}.$$

Here, we set β=2. This fixes the simple equation to be the equation of Riccati or the extended tanh-equation. In addition, we set $s^{*}=3$ and the solved equation becomes

(207) $$\frac{\partial^{2}u}{\partial t^{2}}+\sum_{j=0}^{2}a_{j}{\left(\frac{\partial u}{\partial t}\right)}^{j}-\frac{\partial^{2}u}{\partial x^{2}}=c_{0}+c_{1}u+c_{2}u^{2}+c_{3}u^{3}.$$

For the case of the extended tanh-equation as the simple equation, one obtains a system of nonlinear relationships in Step 4 of the SEsM. A nontrivial solution of this system is

(208) $$\begin{array}{ccc} \theta_{2} & = & 4\frac{a_{2}v^{2}a^{2}}{c_{3}},\\ \theta_{1} & = & 0,\\ \theta_{0} & = & \frac{1}{6a_{2}c_{3}v^{2}}(3v^{2}c_{3}-3c_{3}-2c_{2}a_{2}v^{2}+2(18v^{2}c_{3}^{2}-12c_{1}a_{2}^{2}v^{4}c_{3}+\\ & & 4c_{2}^{2}a_{2}^{2}v^{4}-9v^{4}c_{3}^{2}-9c_{3}^{2}{)}^{1/2}),\\ a_{1} & = & 0,\\ c & = & \frac{1}{8v^{4}aa_{2}^{2}}(18v^{2}c_{3}^{2}-12c_{1}a_{2}^{2}v^{4}c_{3}+4c_{2}^{2}a_{2}^{2}v^{4}-9v^{4}c_{3}^{2}-9c_{3}^{2}{)}^{1/2},\\ c_{0} & = & \frac{1}{108c_{3}^{2}a_{2}^{3}v^{6}}[(18v^{2}c_{3}^{2}-12c_{1}a_{2}^{2}v^{4}c_{3}+4c_{2}^{2}a_{2}^{2}v^{4}-9v^{4}c_{3}^{2}-9c_{3}^{2}{)}^{1/2}(9v^{4}c_{3}^{2}-\\ & & 18v^{2}c_{3}^{2}+9c_{3}^{2}+12c_{1}a_{2}^{2}v^{4}c_{3}-4c_{2}^{2}a_{2}^{2}v^{4})-8c_{2}^{3}a_{2}^{3}v^{6}-108a_{0}c_{3}^{2}a_{2}^{3}v^{6}-\\ & & 108v^{6}c_{3}c_{2}^{2}a_{2}^{2}+324v^{6}c_{3}^{2}c_{1}a_{2}^{2}+108c_{3}c_{2}^{2}a_{2}^{2}v^{4}-324c_{1}a_{2}^{2}v^{4}c_{3}^{2}+\\ & & 270v^{6}c_{3}^{3}-810v^{4}c_{3}^{3}+810v^{2}c_{3}^{3}-270c_{3}^{3}+36c_{1}a_{2}^{3}v^{6}c_{3}c_{2}]. \end{array}$$

The solution of (207) is

(209) $$\begin{array}{ccc} u(\xi ) & = & \frac{1}{6a_{2}c_{3}v^{2}}(3v^{2}c_{3}-3c_{3}-2c_{2}a_{2}v^{2}+2(18v^{2}c_{3}^{2}-12c_{1}a_{2}^{2}v^{4}c_{3}+4c_{2}^{2}a_{2}^{2}v^{4}-\\ & & 9v^{4}c_{3}^{2}-9c_{3}^{2}{)}^{1/2})+\theta_{2}\{-[\frac{1}{8v^{4}a^{2}a_{2}^{2}}(18v^{2}c_{3}^{2}-12c_{1}a_{2}^{2}v^{4}c_{3}+4c_{2}^{2}a_{2}^{2}v^{4}-\\ & & 9v^{4}c_{3}^{2}-9c_{3}^{2}{)}^{1/2}{]}^{1/2}\tanh[(-\frac{1}{8v^{4}a_{2}^{2}}(18v^{2}c_{3}^{2}-12c_{1}a_{2}^{2}v^{4}c_{3}+4c_{2}^{2}a_{2}^{2}v^{4}-\\ & & 9v^{4}c_{3}^{2}-9c_{3}^{2}{)}^{1/2}{)}^{1/2}\left(\xi +\xi_{0}\right)]\}. \end{array}$$

Next, we set β=3. The simple equation, Equation (133), is again reduced to the equation of Bernoulli (134) with k=3. Moreover, we set $s^{*}=3$. Then, from the balance equation, η=4. The solved equation is (207), and in Step 4 of the SEsM, one arrives at the system of nonlinear algebraic relationships. A nontrivial solution of this system is

(210) $$\begin{array}{ccc} \theta_{4} & = & 16\frac{a_{2}v^{2}b^{2}}{c_{3}},\\ \theta_{3} & = & 0,\\ \theta_{2} & = & 16\frac{a_{2}v^{2}ba}{c_{3}},\\ \theta_{1} & = & 0,\\ \theta_{0} & = & \frac{1}{6a_{2}v^{2}c_{3}}\left(8a_{2}^{2}v^{4}a^{2}-2c_{2}a_{2}v^{2}+3v^{2}c_{3}-3c_{3}\right),\\ a_{1} & = & 0,\\ c_{1} & = & -\frac{1}{12c_{3}a_{2}^{2}v^{4}}\left(-4c_{2}^{2}a_{2}^{2}v^{4}+64a_{2}^{4}v^{8}a^{4}+9v^{4}c_{3}^{2}-18v^{2}c_{3}^{2}+9c_{3}^{2}\right),\\ c_{0} & = & -\frac{1}{108c_{3}^{2}a_{2}^{3}v^{6}}(-27c_{3}^{3}+27v^{6}c_{3}^{3}-81v^{4}c_{3}^{3}+81v^{2}c_{3}^{3}+512a_{2}^{6}v^{12}a^{6}+4c_{2}^{3}a_{2}^{3}v^{6}-\\ & & 108c_{0}c_{3}^{2}a_{2}^{3}v^{6}-192c_{2}a_{2}^{5}v^{10}a^{4}-27c_{2}a_{2}v^{2}c_{3}^{2}+54c_{2}a_{2}v^{4}c_{3}^{2}-27c_{2}a_{2}v^{6}c_{3}^{2}). \end{array}$$

The corresponding solution of the solved equation is

(211) $$\begin{array}{ccc} u(\xi ) & = & \frac{1}{6a_{2}v^{2}c_{3}}\left(8a_{2}^{2}v^{4}a^{2}-2c_{2}a_{2}v^{2}+3v^{2}c_{3}-3c_{3}\right)+16\frac{a_{2}v^{2}ba^{2}}{c_{3}}\frac{\exp[2a\left(\xi +\xi_{0}\right)]}{1-b\exp[2a\left(\xi +\xi_{0}\right)]}+\\ & & 16\frac{a_{2}v^{2}b^{2}}{c_{3}}{\left[\frac{a\exp[2a\left(\xi +\xi_{0}\right)]}{1-b\exp[2a\left(\xi +\xi_{0}\right)]}\right]}^{2}, \end{array}$$

for the case b<0, a>0 and

(212) $$\begin{array}{ccc} u(\xi ) & = & \frac{1}{6a_{2}v^{2}c_{3}}\left(8a_{2}^{2}v^{4}a^{2}-2c_{2}a_{2}v^{2}+3v^{2}c_{3}-3c_{3}\right)-16\frac{a_{2}v^{2}ba^{2}}{c_{3}}\frac{\exp[2a\left(\xi +\xi_{0}\right)]}{1+b\exp[2a\left(\xi +\xi_{0}\right)]}+\\ & & 16\frac{a_{2}v^{2}b^{2}}{c_{3}}{\left[\frac{a\exp[2a\left(\xi +\xi_{0}\right)]}{1+b\exp[2a\left(\xi +\xi_{0}\right)]}\right]}^{2}, \end{array}$$

for the case b>0, a<0.

The last example illustrates the application of the SEsM to the case of the use of two simple equations. The set of equations that is discussed contains equations of the nonlinear Schrödinger kind [488]:

(213) $$iq_{t}+aq_{xx}+q\sum_{\begin{array}{c} k=-A\\ k\neq -1 \end{array}}^{B}b_{k}|q{|}^{2k}=0.$$

In order to obtain a solution of Equation (213) one needs the two simplest equations. We skip Step 1 of the SEsM (no transformation of the nonlinearity, as the nonlinearity is a polynomial one). In Step 2 of the SEsM, we can choose a composite function for the solution. The choice is q(x,t)=g(ξ)h(x,t). Here, g(ξ) is a real function (ξ=αx+βt). h(x,t) is a complex function. The two simple equations are for g(ξ) and h(x,t). Below, $h^{*}\left(x,t\right)$ denotes the complex conjugate function of h(x,t). The substitution of the form of q(x,t) in Equation (213) leads to (we denote $g_{\xi }$ as $g^{\prime }$)

(214) $$i\beta g^{\prime }h+igh_{t}+\alpha^{2}ag^{\prime \prime }h+2\alpha ag^{\prime }h_{x}+agh_{xx}+\left(gh\right)\sum_{\begin{array}{c} k=-A\\ k\neq -1 \end{array}}^{B}g^{2k}{\left(hh^{*}\right)}^{k}=0.$$

Here, $g_{\xi }$$g^{\prime }$ denotes $g_{\xi }$. The simple equation for h(x,t) is $h_{\zeta }=ih,\;\;\zeta =\kappa x+\omega t+\theta$. The solution is h(ζ)=exp(iζ)=exp[i(κx+ωt+θ)]. This choice of *h* leads to the equation for g(ξ)

(215) $$\alpha^{2}ag^{\prime \prime }+\left(2\alpha \kappa a+\beta \right)ig^{\prime }-\left(\omega +\kappa^{2}a\right)g+g\sum_{\begin{array}{c} k=-A\\ k\neq -1 \end{array}}^{B}b_{k}g^{2k}=0.$$

g(ξ) must be a real function and because of this, β=−2ακa. We substitute this in (215) and multiply the result by $g^{\prime }$. What is obtained is integrated with respect to ξ. The result is

(216) $$\alpha^{2}ag^{\prime 2}-\left(\omega +\kappa^{2}a\right)g^{2}-c+\sum_{\begin{array}{c} k=-A\\ k\neq -1 \end{array}}^{B}\frac{b_{k}}{k+1}g^{2k+2}=0.$$

*c* is a constant of integration. We introduce a new variable $u=g^{\sigma }$. Here, σ is a parameter. The equation for u(ξ) becomes

(217) $$u^{\prime 2}=\frac{\sigma^{2}\left(\omega +\kappa^{2}a\right)}{\alpha^{2}a}u^{2}+\frac{\sigma^{2}c}{\alpha^{2}a}u^{2(\sigma -1)/\sigma }-\sum_{\begin{array}{c} k=-A\\ k\neq -1 \end{array}}^{B}\frac{\sigma^{2}b_{k}}{\alpha^{2}a\left(k+1\right)}u^{2(k+\sigma )/\sigma }.$$

Equation (217) has various specific cases. Three of them are:

1. σ=1, k=0,…,B. Then, (217) becomes the following equation:
  (218) $$u^{\prime 2}=\sum_{k=0}^{B}c_{k}u^{2(k+1)},\;\;\;c_{0}=\frac{\omega +\kappa^{2}a-b_{0}}{\alpha^{2}a};\;\;c_{k}=-\frac{b_{k}}{\alpha^{2}a\left(k+1\right)},\;k=1,\ldots ,B.$$
2. σ=2, k=−2,…,B. Then, (217) becomes
  (219) $$\begin{array}{c} u^{\prime 2}=\sum_{k=-2}^{B}c_{k}u^{k+2},\;\;\;c_{-2}=-\frac{b_{-2}}{\alpha^{2}a};\;\;c_{-1}=\frac{4c}{\alpha^{2}a};\;\;c_{0}=\frac{4\omega +4\kappa^{2}a-4b_{0}}{\alpha^{2}a};\\ c_{k}=-\frac{4b_{k}}{\alpha^{2}a\left(k+1\right)},k=1,\ldots ,B. \end{array}$$
3. c=0, σ: arbitrary positive integer that is different from 1, B=Dσ, k=nσ, n=−1,…,D. Then, (217) becomes
  (220) $$\begin{array}{c} u^{\prime 2}=\sum_{n=-1}^{D}c_{n}u^{2(n+1)},\;\;\;c_{-1}=-\frac{\sigma^{2}b_{-\sigma }}{\alpha^{2}a\left(1-\sigma \right)},\;\;c_{0}=\frac{\sigma^{2}\left(\omega +\kappa^{2}a-b_{0}\right)}{\alpha^{2}a},\\ c_{n}=-\frac{\sigma^{2}b_{n\sigma }}{\alpha^{2}a\left(\sigma n+1\right)},n=1,\ldots ,D. \end{array}$$

The solutions of (218)–(220) can be expressed by the special function $V_{\vec{a}}\left(\xi ;q,l,m\right)$ from [486]. Remember that this special function is the solution of ${\left(\frac{d^{q}u}{d\xi^{q}}\right)}^{l}=\sum_{j=0}^{m}a_{j}u^{j}$. Here, *q*, *l*, and *m* are positive integers and $\vec{a}=\left(a_{0},\ldots ,a_{m}\right)$. The solutions of (213) are:

1. σ=1, k=0,…,B.
  (221) $$q\left(x,t\right)=V_{{\vec{a}}_{1}}\left(\xi ;1,2,2B+2\right)\exp\left[i\left(kx+\omega t+\theta \right)\right].$$
  Here, ${\vec{a}}_{1}=\left(a_{0},\ldots ,a_{2B+2}\right)$ and $a_{0}=0$, $a_{2i}=c_{i-1}$, $a_{2i-1}=0$, i=1,…,B+1.
2. σ=2, k=−2,…,B.
  (222) $$q\left(x,t\right)={\left[V_{{\vec{a}}_{2}}\left(\xi ;1,2,B+2\right)\right]}^{1/2}\exp\left[i\left(kx+\omega t+\theta \right)\right].$$
  Here, ${\vec{a}}_{2}=\left(a_{0},\ldots ,a_{B+2}\right)$ and $a_{i}=c_{i-2}$, i=0,…,B+2.
3. c=0, σ: an arbitrary positive integer that is different from 1, B=Dσ, k=nσ, n=−1,…,D.
  (223) $$q\left(x,t\right)={\left[V_{{\vec{a}}_{3}}\left(\xi ;1,2,2D+2\right)\right]}^{1/\sigma }\exp\left[i\left(kx+\omega t+\theta \right)\right].$$
  Here, ${\vec{a}}_{3}=\left(a_{0},\ldots ,a_{2D+2}\right)$ and $a_{2i}=c_{i-1}$, $a_{2i+1}=0$, i=0,D+1.

The above solutions can be expressed by the specific case of the *V*-functions for the selected values of the parameters. One example is for the solutions given by Equation (222). These solutions are:

1. Case B=0. Equation (213) is the solution, which is
  (224) $$q\left(x,t\right)={\left\{\cosh\left[\pm {\left(b_{-2}/a\right)}^{1/2}\left(x-2\kappa at\right)\right]\right\}}^{1/2}\exp\left\{i\left[\kappa x+\frac{1}{4}\left(b_{-2}-4\kappa^{2}a+4b_{0}\right)t+\theta \right]\right\}.$$
2. Case B=1. Equation (213) is
  (225) $$iq_{t}+aq_{xx}+b_{-2}q|q{|}^{-4}+b_{0}q+b_{1}q|q{|}^{2}=0.$$
  The solution of Equation (225) is
  (226) $$q\left(x,t\right)={\left\{℘\left[\pm {\left(-b_{1}/\left(2a\right)\right)}^{1/2}\left(x-2\kappa at\right);0,2b_{-2}/b_{1}\right]\right\}}^{1/2}\exp\left\{i\left[\kappa x+\left(b_{0}-\kappa^{2}a\right)t+\theta \right]\right\}.$$
  Here, *℘* is the elliptic function of Weierstrass.
  Case B=2. Equation (213) is
  (227) $$iq_{t}+aq_{xx}+b_{-2}q|q{|}^{-4}+b_{0}q+b_{1}q|q{|}^{2}+b_{2}q|q{|}^{4}=0,$$
  The solution is expressed by the Jacobi elliptic functions [488].

The list of examples of the application of the SEsM could continue. For more applications, see, for example, [460,463,467,468,469,470,484,485,487,488,508,509,510].

## 9. The SEsM and Other Methods for Obtaining Specific Exact Solutions of Nonlinear Nonintegrable Differential Equations

An interesting direction for future research is the relationship between the SEsM and other methods for obtaining exact solutions of nonlinear differential equations. We have shown above that by using the appropriate composite function of Step 2 of the SEsM and simple equations for exponential functions, the SEsM can be connected to the method of Hirota and the Inverse Scattering Transform method. These two methods are often used for obtaining exact solutions of integrable nonlinear differential equations. The situation with respect to the methods for obtaining particular solutions of nonintegrable nonlinear differential equations is as follows. It was shown that the SEsM contains as specific cases [511] the Jacobi Elliptic Function Expansion method [512], F-Expansion method [513,514,515], Modified Simple Equation method [516], Trial Function method [517,518], General Projective Riccati Equations method [519], and First Integral method [520]. Other methods that demonstrate specific cases of the SEsM are [495] the Homogeneous Balance method [521,522,523,524] and the Auxiliary Equation method [525]. The list continues [466] with the G’/G method [526,527], Exp-function method [528], tanh method [529], and the method of the Fourier series. We note that the SEsM can also deal with differential equations containing fractional derivatives [465]. The list can be continued and we present here the following conjecture.

**Conjecture** **1.** *Any method for obtaining exact analytical solutions of nonintegrable nonlinear differential equations is a specific case of the SEsM.*

The motivation for this conjecture is as follows. The SEsM is based on known solutions of simple equations and the solution of the more complicated solved equation is constructed as a composite function of these solutions. Any method for obtaining exact analytical solutions of nonlinear differential equations has to construct these solutions using known functions. These known functions are the solutions of the simple equations. The solution of the solved equation is a function of these known functions. This is the composite function used in the SEsM. Then, if one wants to find a method for obtaining the exact solution of a nonlinear differential equation that is not a specific case of the SEsM, one has to do one of the following things:

1. Use the forms of the known functions that are not solutions of any simple equations. This will be extremely difficult as one has to use functions that are not solutions of differential equations.
2. Construct the searched solution as a function of the known functions, which is not a composite function. This is also an extremely difficult task.

## 10. Concluding Remarks

In this review article, we discuss an effective method for obtaining exact analytical solutions of nonlinear differential equations, the Simple Equations Method (SEsM). Through a relatively large overview of the literature, we show that the research area connected to the search for exact solutions of nonlinear differential equations is of much interest because these equations are used in the mathematical models of numerous complex systems, and the mathematical aspect of the search of exact solutions is also quite interesting. The SEsM has a simple algorithm based on the appropriate transformation of the nonlinearity of the solved equation (if necessary), and then the solution is constructed as a composite function of solutions of simpler differential equations. The SEsM has four steps. In Step 1, one may use a transformation to convert the nonlinearity of the solved equation to a more treatable kind of nonlinearity. A treatable kind of nonlinearity is polynomial nonlinearity. Step 2 of the SEsM is connected with the use of a composite function in order to build the solution of the solved equation. We show that the composite function favors the occurrence of a certain class of simple equations that define a special function, the *V*-function. This function contains as specific cases numerous elementary and special functions and leads to the occurrence of systems of polynomials in the process of the application of the SEsM to the solved equation. The SEsM transforms the solved equation into a sum of functions multiplied by some coefficients, which are relationships between the parameters of the solved equation and the parameters of the solution. The setting of these coefficients to 0 leads to a system of nonlinear algebraic equations. In order to obtain a nontrivial solution of the solved equation, this system of nonlinear algebraic relationships must be balanced. This leads to additional relationships called balance equations. The balance equations are important as they may lead to a fixation of the form of the simple equation and the form of the composite function for the solution of the solved equation. Thus, the first three steps of the SEsM convert the solved equation to a system of nonlinear algebraic relationships. The solution of this system is the task of Step 4 of the SEsM. Each obtained nontrivial solution of the system of nonlinear algebraic relationships leads to a nontrivial solution of the solved equation.

We note that in general, the SEsM is constructed to be able to solve systems of nonlinear differential equations. Above, we presented the version of the method for the solution of one nonlinear differential equation. We demonstrated that this version of the SEsM is connected to well-known methods for obtaining multisoliton exact analytical solutions of integrable differential equations, the Inverse Scattering Transform method, and the method of Hirota. We showed how the SEsM led to the equation of Gelfand–Levitan–Marchenko for the case of the Korteweg-de Vries equation and to the relationships of Zakharov and Shabat for the case of the nonlinear Schrödinger equation. We discussed numerous transformations of nonpolynomial nonlinearities to polynomial nonlinearities in Step 1 of the SEsM. The need to use composite functions arose naturally in the SEsM as the solution had to be constructed as a composite function of the solution of the simple equations. This led to the occurrence of the Faa di Bruno formula for the derivatives of the composite functions in the process of the application of the SEsM.

The use of composite functions in the SEsM favors simple equations with polynomial nonlinearity. Thus, simple equations, such as the equations of Bernoulli and Riccati and the equations for the elliptic functions of Weierstrass and Jacobi, are often used in the SEsM. The connection between the choice of the simple equation and the form of the composite function for the solution of the solved equation was discussed with special attention in the text. Numerous examples of the application of the SEsM were presented. These applications illustrated the methodology and presented exact analytical solutions of many equations obtained over the years with the use of the SEsM in our research. Special attention was given to the application of the SEsM to differential equations arising in connection with the SIR model of epidemic spreading. The obtained solutions can be used for modeling COVID-19 waves. Other examples were connected to exact analytical solutions of the equations of Fisher and Huxley, as well as the solutions of the generalized Camassa–Holm, Swift–Hohenberg, and Rayleigh equations. Special attention was also devoted to obtaining exact solutions of nonlinear differential equations such as the nonlinear equation of Schrödinger. The reason is that we showed the application of the SEsM for the case of two simple equations in the process of obtaining exact analytical solutions of these equations.

As we have shown above, the methodology of the SEsM is effective. It is connected to well-known methods for obtaining exact multisoliton solutions of nonlinear differential equations and contains as specific cases many methods for obtaining specific exact analytical solutions of nonintegrable equations. Future research on the SEsM will be in two directions: (i) an extension of the area of the application of the methodology by treating additional classes of nonlinearities and equations; and (ii) the application of the methodology to systematic methods for obtaining exact solutions of nonlinear differential equations.

## Data Availability

Not applicable.
